# Microbiome Signatures and Inflammatory Biomarkers in Culture-Negative Neonatal Sepsis

**DOI:** 10.3390/applmicrobiol5030057

**Published:** 2025-06-24

**Authors:** Morcos Hanna, Shixia Huang, Matthew Ross, Anaid Reyes, Dimuthu Perera, Anil Surathu, Sara Javornik Cregeen, Joseph Hagan, Mohan Pammi

**Affiliations:** 1 Section of Neonatology, Department of Pediatrics, Baylor College of Medicine and Texas Children’s Hospital, Houston, TX 77030, USA; 2 Department of Molecular and Cellular Biology, Baylor College of Medicine, Houston, TX 77030, USA; 3 Department of Education, Innovation & Technology, Baylor College of Medicine, Houston, TX 77030, USA; 4 Alkek Center for Metagenomics and Microbiome Research, Department of Molecular Virology and Microbiology, Baylor College of Medicine, Houston, TX 77030, USA

**Keywords:** microbiome, neonatal sepsis, culture-negative sepsis

## Abstract

Overuse of antibiotics is a concern in ‘culture-negative sepsis’ but it is unclear whether this is due to infection with viruses, fungi or other microbes that are not easily cultured, or whether it results from inflammatory processes. In a prospective study, we enrolled 50 preterm neonates with culture-positive sepsis (CP), culture-negative sepsis (CN), and asymptomatic preterm controls (CO). The microbiome of stool, skin, and blood, including bacterial, viral and fungal components and serum cytokine profiles were evaluated. The microbiome alpha or beta diversity did not differ between CN and CO groups. A MaAsLin analysis revealed increased relative abundances of specific bacterial and fungal genera in stool and skin samples in the CN group compared to CO. The virome analysis identified 24 viruses from skin samples, but they were not statistically different among the three groups. The cytokine and chemokine biomarker profiles were elevated in the CP group but were not statistically different between the CN and CO groups. Although the CN group had a longer hospital stay and higher BPD rates than the controls in unadjusted analyses, these differences were not significant after adjusting for gestational age and birth weight. The CN infants demonstrated microbial shifts without systemic immune activation or significantly worse clinical outcomes, supporting the rationale for discontinuing antibiotics in the absence of positive cultures.

## 1. Introduction

Sepsis is a leading cause of neonatal morbidity and mortality, often presenting with non-specific clinical signs and symptoms [1]. The term ‘culture-negative sepsis’ is often used to describe neonates who are deemed symptomatic and treated with antibiotics when blood cultures are negative, but its existence is debated [2]. It is unclear whether ‘culture-negative sepsis’ is related to infection with viruses, fungi, or other difficult-to-culture microbes, inadequate blood volume inoculation for cultures, or inflammatory processes. Reports suggest that antibiotic exposure occurs up to 10 times more for neonates with culture-negative sepsis than for those with culture-proven sepsis [3,4]. The unintended harm caused by prolonged or unnecessary antibiotic exposure in neonates includes increased risk of developing fungal infections, necrotizing enterocolitis (NEC), bronchopulmonary dysplasia (BPD), and death [5,6].

Understanding the microbial and inflammatory landscape of these infants has become increasingly feasible with advances in next-generation sequencing (NGS), which have expanded our view of the human microbiome, including bacterial, fungal, and viral components, and their role in health and disease [7]. In neonates, the microbiome plays a key role in immune development and disease susceptibility during this critical window of immune and physiological maturation [8,9]. While most studies focus on bacterial communities, recent research highlights the relevance of the mycobiome and virome in human health and disease [10,11].

These insights are especially relevant in neonates, whose microbiome is rapidly established after birth and shaped by factors such as delivery mode, feeding type, and environmental exposure [12–14]. For example, breastfed and vaginally delivered infants tend to have different microbial communities than those born by cesarean section or fed formula. The neonatal virome is minimal at birth but rapidly develops, starting with bacteriophages and later including eukaryotic viruses that infect human cells, with their composition influenced by feeding type [15,16]. Additionally, there is emerging evidence that microbial colonization may begin in utero, with low-abundance but metabolically active bacteria and viruses detected in fetal gut tissues [17]. However, the clinical significance of in utero colonization remains under investigation.

We hypothesized that preterm neonates with clinical signs of sepsis would exhibit distinct microbiome profiles and elevated pro-inflammatory cytokine and chemokine levels compared to asymptomatic controls, with potential differences between the culture-positive and culture-negative sepsis groups. Our objectives were as follows: (1) to characterize the diversity and composition of the microbiome (bacterial, viral, and fungal) in the blood, skin, and stool of preterm neonates across three groups: culture-positive sepsis, culture-negative sepsis, and controls; and (2) to evaluate associations between cytokine/chemokine profiles, sepsis groups, and neonatal outcomes.

## 2. Materials and Methods

### 2.1. Study Design and Participants

This was a prospective pilot study conducted in the level III and IV neonatal intensive care units at Texas Children’s Hospital. This study was approved by the Institutional Review Board at Baylor College of Medicine, Houston, Texas (H-43505) on 25 March 2021. All preterm infants (born less than 37 weeks’ gestation) who were admitted to Texas Children’s Hospital’s NICU between May 2021 and May 2023 were eligible to participate. Exclusion criteria included major congenital malformations and suspected or confirmed immune deficiencies. Sample size calculations were based on pilot data from prior bacterial microbiome research [18,19]. The mean ± standard deviation of the Shannon Diversity Index (SDI) for non-infected patients was found to be 2.30 ± 0.74. Thus, a sample size of 20 patients per group was targeted to provide 80% statistical power to detect an effect size of 1.00 (in SDI) at the 5% significance level.

This study included three distinct patient groups, based on clinical presentation and blood culture results: the culture-positive (CP) group, comprising symptomatic infants with clinical signs suggestive of sepsis and positive blood cultures; the culture-negative (CN) group, including symptomatic infants with signs of sepsis but negative blood cultures; and the asymptomatic control (CO) group, consisting of infants without clinical signs of sepsis, from whom blood, serum, stool, and skin samples were collected for research purposes. The control infants were matched primarily based on postnatal age to account for the rapid development of the neonatal microbiome and virome. Samples from control patients were obtained during clinically indicated blood draws whenever possible to minimize additional procedures.

The infants in the culture-positive and culture-negative sepsis groups were enrolled during late-onset sepsis evaluations (>72 h of age), prior to the initiation of antibiotic therapy. At our institution, there are no standardized criteria for initiating a sepsis workup in preterm infants; instead, evaluations are conducted at the discretion of the clinical team based on signs such as temperature instability, apnea, bradycardia, increased respiratory support, and feeding intolerance. After informed consent was obtained, blood and stool samples, as well as skin swabs, were collected and stored at −80 °C until processing. Due to infrequent stool passage, stool sample collection was often delayed; nonetheless, 48 of 50 stool samples were successfully collected and included in the analysis, offering a broad view of gut microbiome composition during suspected sepsis. Two patients were excluded from stool analysis due to logistical challenges. Enrollment continued until a sufficient number of infants with culture-positive sepsis had been included. Cytokine and chemokine profiles were also assessed at the time of evaluation, and detailed maternal and neonatal clinical metadata were collected, including preterm labor, maternal antibiotic exposure, mode of delivery, gestational age, 5 min Apgar score, prior neonatal antibiotic use, and days requiring oxygen support.

### 2.2. Bacterial and Fungal Microbiome Evaluation

The microbiome, mycobiome, and virome of stool, skin, and blood samples were evaluated by methods established at the Center for Metagenomics and Microbiome Research (CMMR) at Baylor College of Medicine. Total genomic DNA and RNA were extracted using the Qiagen MagAttract PowerMicrobiome DNA/RNA kit (stool, skin) and the Qiagen DNeasy Blood and Tissue kit (blood) according to the manufacturer’s instructions, with the exception of the skin swab samples that were eluted in 40 μL. 16S rRNA and ITS2 gene sequencing methods were adapted from those developed for the NIH-Human Microbiome Project and the Earth Microbiome Project [20–22]. The 16S rDNA v4 hypervariable region was amplified by PCR using region-specific primers (515F: GTGCCAGCMGCCGCGGTAA, 806R: GGACTACHVGGGTWTCTAAT). The ITS2 region was amplified by PCR using primers (ITS3: GCATCGATGAAGAACGCAGC, ITS4: TCCTCCGCTTATTGATATGC). Primers used for amplification contained adapters for Illumina platform sequencing and single-index barcodes incorporated into the reverse primer allowing the PCR products to be pooled and sequenced directly. Barcoded libraries were sequenced on the Illumina MiSeq platform using the 2 × 250 bp or 2 × 300 bp paired-end protocol for 16Sv4 and ITS2 amplicons, respectively. Raw data files in binary base call (BCL) format were converted into FASTQs and demultiplexed based on the single-index barcodes using the Illumina ‘bcl2fastq’ software, version v2.20.

The demultiplexed read pairs underwent an initial quality filtering using bbduk.sh (BBMap, version 38.82) [23], removing Illumina adapters, PhiX reads, and reads with a Phred quality score < 15 and length < 100 bp after trimming. Quality-controlled reads were merged using bbmerge.sh with amplicon-specific merge parameters. The merged reads were further filtered via vsearch [24] using parameters optimized for the appropriate amplicon type. All reads were combined into a single FASTA file and processed via the UPARSE algorithm [25]. Abundances were recovered by mapping the demultiplexed reads to the representative sequences file, creating a feature table in biom format, and removing the chimeric reads. The generated representative sequences were mapped against an optimized version of the latest current SILVA Database (Release 138.2) [26,27], containing only sequences from the V4 region of the 16S rRNA gene, or the UNITE Database [28] for ITS2 amplicons, to determine taxonomies using the usearch70 ‘usearch_global’ function, with the identity threshold set to 97%. The phylogeny information for the 16Sv4 data contained in the biom file was generated by aligning the centroid sequences with mafft [29] and creating a tree via FastTree [30]. The biom file was summarized, recording the number of reads per sample, and merged with a file that was generated for the overall read statistics, to produce a final summary file with read statistics and taxonomy information. Output files were analyzed using the Agile Toolkit for Incisive Microbial Analyses (ATIMA, https://atima.research.bcm.edu). The pairwise differential abundance analysis of Amplicon data (16Sv4 and ITS2) among the three groups of interest (culture-positive, culture-negative, and controls) was performed using Microbiome Multivariable Association with Linear Models2 (MaAsLin2) [31].

### 2.3. Virome Evaluation

RNA extracts were converted to cDNA using Protoscript II First Strand cDNA Synthesis Kit (New England Biolabs Inc., Ipswich, MA, USA), NEBNext Ultra II Non-Directional RNA Second Strand Module (New England Biolabs Inc.), and Random Primer 6 (New England Biolabs Inc.). A total of 25 ng of the cDNA and DNA mix was used for library construction using the Twist EF 2.0 library preparation kit (Twist) with Twist UDI Index Kit. The libraries were pooled, a maximum of 16 samples per pool, at equal mass, to a total of 1500 ng per pool. The Twist Comprehensive Viral Research Panel (Twist Biosciences, San Francisco, CA, USA) was used to hybridize the probes at 70 °C for 16 h. The post-capture pool was further PCR-amplified for 12 cycles, and final libraries were sequenced on the Illumina NovaSeq 6000 platform, to generate 2 × 150 bp paired-end reads. Following sequencing, raw data files in BCL format were converted into FASTQs and demultiplexed based on the dual-index barcodes using Illumina ‘bcl2fastq’ software.

Virome taxonomic profiling was performed using EsViritu (v0.2.3) [32]. The preprocessed reads were aligned to the Virus Pathogen Database (v2.0.2) via minimap2 [33], and alignments were filtered by CoverM (https://github.com/wwood/CoverM, accessed on 5 March 2023) to require at least 90% average identity across 50% of the read length. Virus genomes/segments with reads covering at least 1000 nucleotides or 50% of the genome/segment length were considered preliminary detections. Consensus sequences from this preliminary set of detected virus genomes/segments were extracted using samtools consensus [34] and compared to each other pairwise using anicalc. The longest consensus sequence in each cluster was kept as the final sequence. The fastq reads were then re-aligned to a smaller database of only the references corresponding to final sequences with the same parameters for minimap2/CoverM, resulting in final detections. The metric RPKMF was calculated as (reads per kilobase of reference genome)/(million reads passing filtering). Finally, per-sample abundance and coverage metrics and taxonomic information were gathered in tabular format and merged into a single table for downstream processing. Esviritu virome outputs were analyzed using microeco [35], an R package, version v1.15.0 to perform microbiome analysis and generate visualizations. The Shannon alpha diversity index was calculated, and pairwise comparisons of groups were performed using the Wilcoxon rank-sum test. For beta diversity, Bray–Curtis dissimilarity matrices were generated, and PERMANOVA tests were conducted to compare groups. Pairwise differential abundance analysis of the Virome data between the groups was performed using MaAsLin2. The estimated counts table generated by EsViritu was used as input, and the analysis was conducted with the following parameters: the minimum abundance was set to 1, the minimum prevalence to 0.3, the maximum significance threshold to 0.25, the normalization method to Total Sum Scaling (TSS), and the analysis method employed was Compound Poisson Linear Models (CPLMs).

### 2.4. Evaluation of Cytokine and Chemokine Profiles

Blood was assayed using the ProcartaPlex^™^ Human Immune Monitoring Panel 65-Plex (Cat#: EPX650–10065-901). The analysis was performed at the Antibody-Based Proteomics Core at Baylor College of Medicine. Following calibration and validation of the instrument, reagents were prepared as directed by the manufacturer (Thermo Fisher, Waltham, MA, USA). Sample and beads were incubated at room temperature on shaker for 2 h. Sufficient beads were obtained for standards, QCs, and samples. A Logistic-5PL regression type was used to construct the standard curve of all the analytes. Standard points that had a negative MFI-Bkgd value or had an (Obs/Exp) × 100 value outside of the 80–120% were designated as outliers. We also evaluated the cytokine and chemokine profiles in relation to the effects of tissue inflammatory injury such as BPD, retinopathy of prematurity (ROP), and development of NEC.

### 2.5. Statistical Analyses and Data Interpretation

The mean and median were used to quantify central tendency of the groups’ SDI and OTUs, while the standard deviation and range were used to summarize variability. If the outcome was normally distributed in all three groups, as determined by the Shapiro–Wilk test, an analysis of variance (ANOVA) was used to compare the groups’ SDI and OTUs, with ‘Tukey’s Honestly Significant Differences’ test used for post-hoc analysis to determine which pairs of groups were significantly different. If the assumption of normality was violated, the comparisons were conducted using the Kruskal–Wallis test, and post-hoc analyses were performed using the Wilcoxon rank-sum test, with Bonferroni-adjusted *p*-values used for pairwise comparisons. Furthermore, we assessed the cytokine and chemokine profiles in relation to the effects of tissue inflammatory injury such as BPD, ROP, and development of NEC. Logistic regression analysis was used to examine associations of inflammatory markers with binary clinical outcomes, while linear regression and correlation analysis were used for the ‘length of hospital stay’ outcome.

## 3. Results

**Figure 1.**
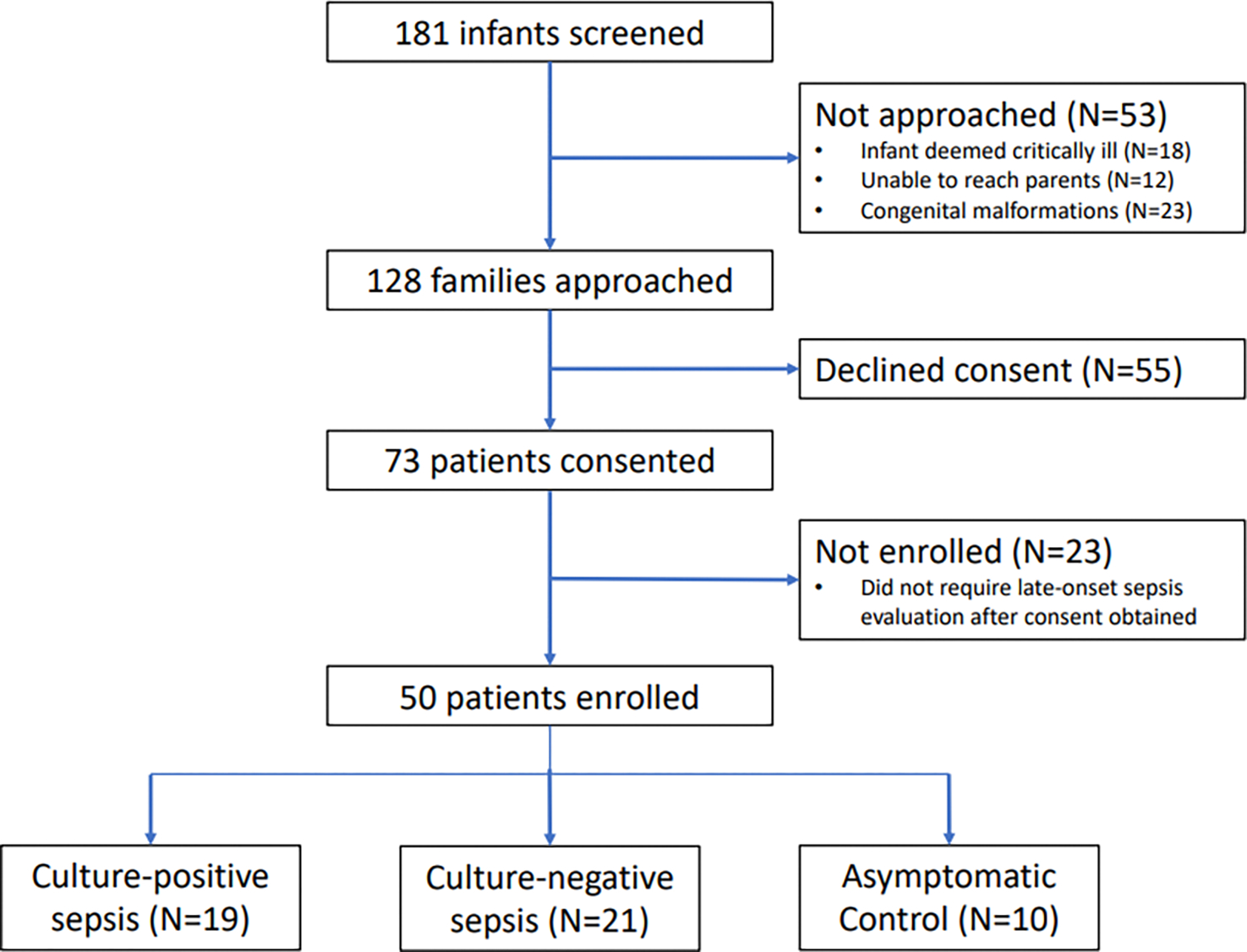
Enrollment process of the prospective preterm cohort with suspected sepsis and controls.

A total of 50 preterm neonates were enrolled in this research study. The three study groups comprised of 19 patients with culture-positive sepsis (CP), 21 patients with culture-negative sepsis (CN), and 10 asymptomatic preterm neonates as controls (CO) (Figure 1). The characteristics of the enrolled patients are compared in Table 1. The median time from sepsis evaluation to stool sample collection was 1 day (interquartile range [IQR] 3 days, range 0–17 days). Delays in stool collection occurred primarily due to infrequent stooling in preterm infants, which is common in this population. As a result, some samples were collected after antibiotic exposure had already occurred, which may have influenced the microbial composition of the stool at the time of analysis.

### 3.1. Microbiome Profiles in Preterm Infants

**Figure 2.**
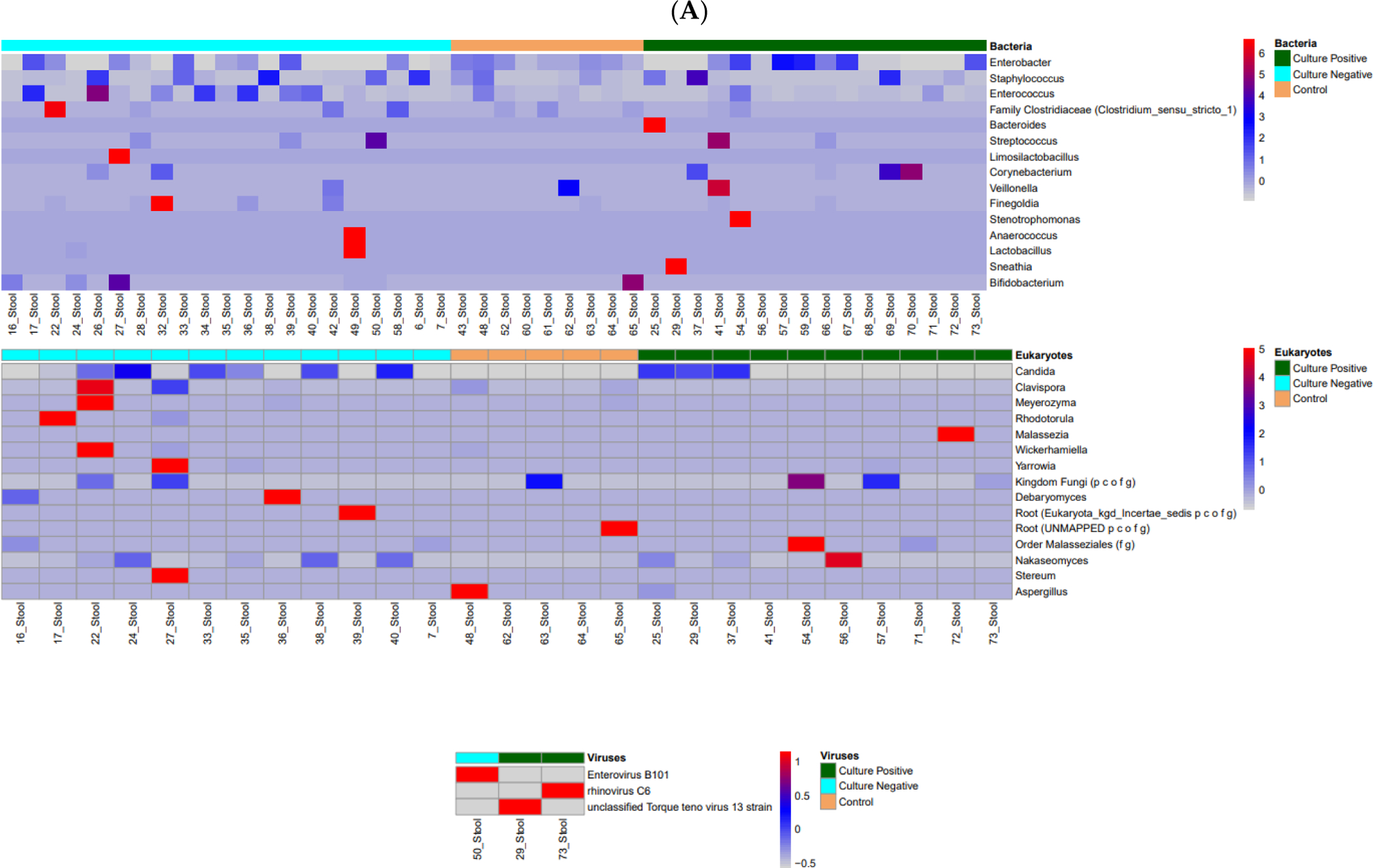

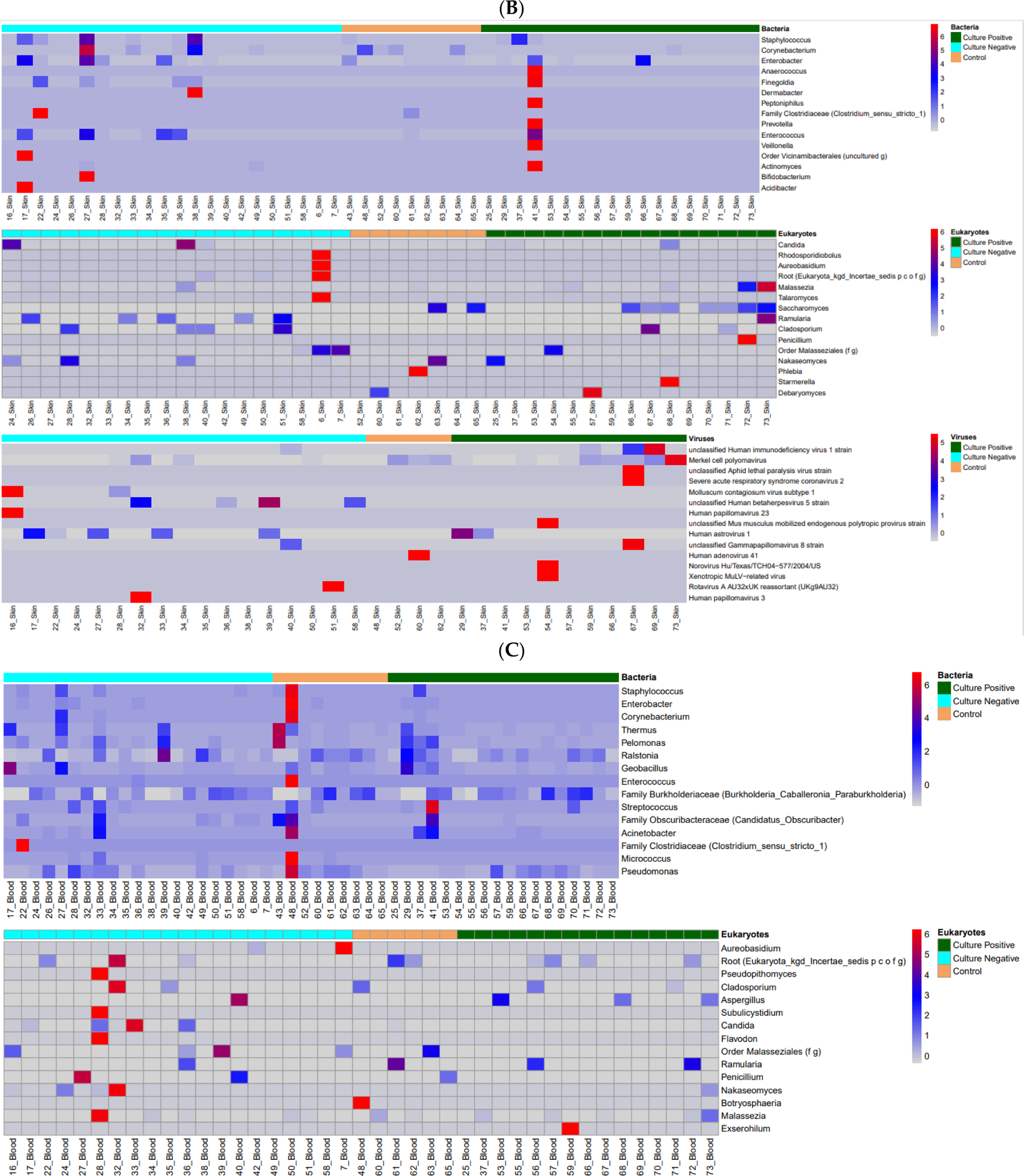
Heatmap of the stool (**A**), skin (**B**), and blood (**C**) microbiome profiles in patients with culture-positive sepsis, culture-negative sepsis, and controls. Sub-panels depict bacteria, eukaryotes, and viruses ((**A**) and (**B**) only). The top row of the heatmap indicates the patient groups: light blue indicates the culture-negative group, orange indicates the controls, and dark green indicates the culture-positive group. Th red–blue legend scale represents the increase or decrease in microbial profiles. The heat maps provide a visual representation of the microbial profiles, and statistical associations are presented in Table 2.

We evaluated the stool, skin, and blood microbiome in the enrolled cohort, including 16S evaluation for the bacterial microbiome, ITS2 evaluation for the mycobiome, and viral detection. The stool microbiome and mycome evaluation did not show significant differences in alpha diversity or beta diversity among the three groups of neonatal sepsis (Supplementary Figures S2 and S5). A heatmap of the stool microbial composition is represented in Figure 2A. The MaAsLin2 analysis revealed differences in relative abundances of specific microbial genera between the culture-negative and control groups (Table 2). For bacterial genera, 16S data showed that the relative abundances of *Enterobacter*, *Streptococcus*, *Corynebacterium*, and *Enterococcus* were significantly different between the culture-negative and control groups. Similarly, the ITS2 analysis identified significant differences in the relative abundances of fungal taxa, including *Candida*, *Nakaseomyces*, and *Saccharomyces*, in the stool between culture-negative and control groups. Only three patients had viruses identified in the stool (two in the CP group and one in the CN group), limiting the ability to draw meaningful conclusions.

There were no significant differences in the alpha or beta diversity among the three groups with respect to the skin bacterial or fungal microbiome (Supplementary Figures S3 and S6). A heatmap of the skin microbial composition is represented in Figure 2B. MaAsLin2 analysis revealed specific taxa with differential relative abundances (Table 2). For bacteria, the 16S data identified significant differences in the relative abundances of *Staphylococcus*, *Haemophilus*, and *Streptococcus* between the groups. Similarly, the ITS2 fungal analysis revealed significant differences in the relative abundances of *Candida* and *Saccharomyces*. We identified most of the viruses from the skin (32 from the skin, three from the stool, and one from the blood, among the 50 patients enrolled); however, there were no statistically significant differences in the diversity of skin viruses among the groups (Supplementary Figure S7). The identified viruses are listed in the supplemental file, with 24 viruses identified across 32 samples.

We also evaluated the circulating blood microbiome to test our hypothesis that circulating microbial DNA may incite inflammation and clinically present as culture-negative sepsis. A heatmap of the blood microbial composition is represented in Figure 2C. Although we found bacterial and fungal DNA in the blood, there were no significant differences among the groups or specifically, between the culture-negative group and controls (Table 2 and Supplementary Figure S4).

### 3.2. Systemic Cytokine and Chemokine Biomarkers

**Figure 3.**
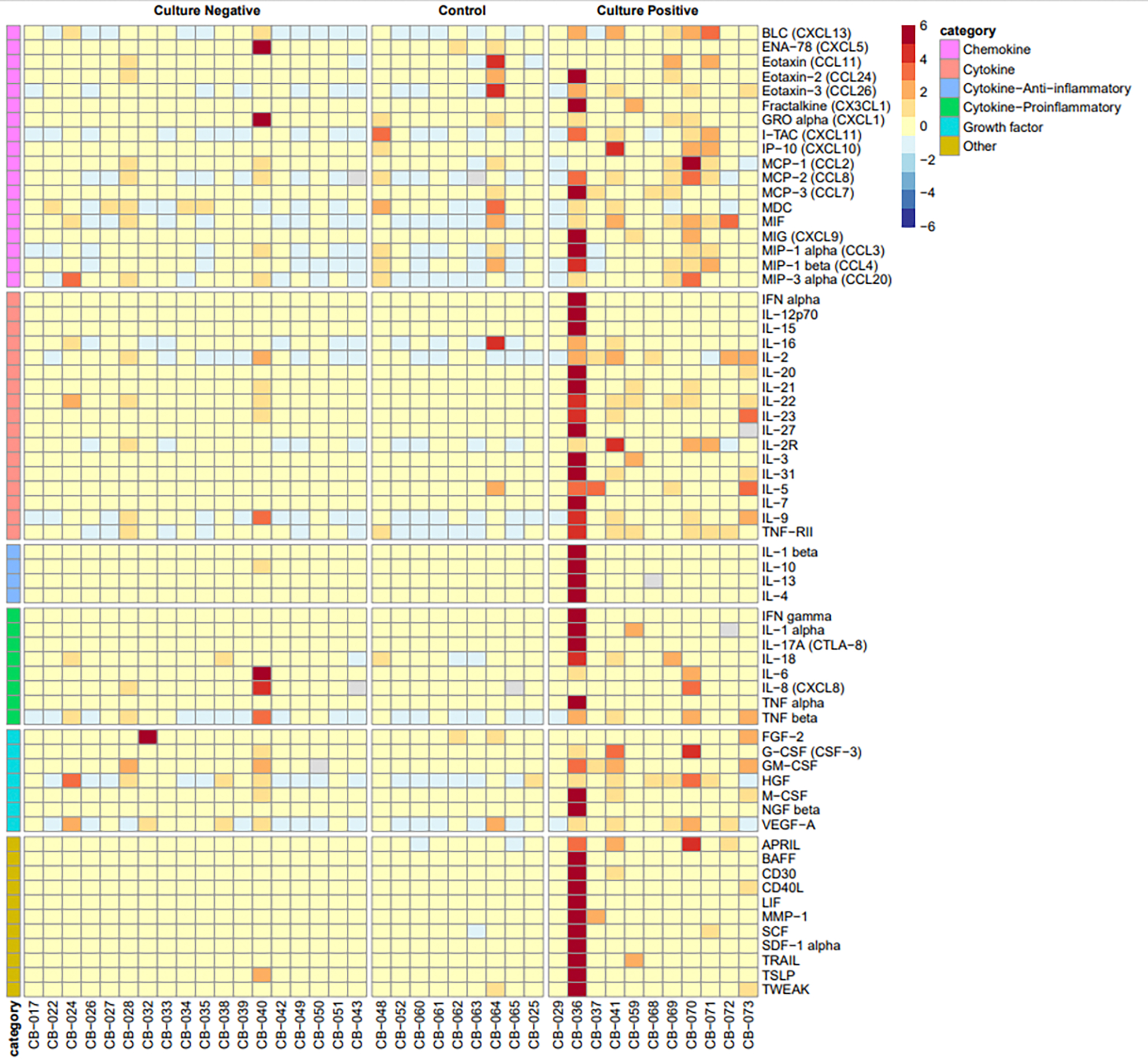
Heatmap of cytokine and chemokine biomarker profiles in patients with culture-positive sepsis, culture-negative sepsis and controls. The type of cytokine or chemokine is represented in the first column. The labels on the top of the heatmap indicate the patient groups, the culture-positive group, the culture-negative group, and the controls. The red–blue legend scale represents the increase or decrease in cytokine and chemokine biomarker profiles. Increased biomarker profiles are seen in the culture-positive group, but there were no significant differences in cytokine and chemokine profiles between the culture-negative and control groups.

Cytokine and chemokine analysis was possible for only 38 of the enrolled patients (CN: *n* = 18, CO: *n* = 9, CP: *n* = 11). Comparing the inflammatory markers among the study groups, we observed distinct patterns. The preterm infants with CP sepsis exhibited significantly elevated levels of 24 inflammatory markers compared to the CN sepsis group and 32 inflammatory markers compared to asymptomatic healthy controls (Figure 3 and Table 3). These findings suggest a heightened systemic inflammatory response in culture-positive sepsis, in response to infection. In contrast, none of the inflammatory cytokines or chemokines were statistically different between the CN and CO groups (Table 3).

### 3.3. Preterm Sepsis Groups and Neonatal Outcomes

The differences in the clinical outcomes between the sepsis groups and controls are represented in Table 4. Among the outcomes evaluated, ROP, BPD, and length of stay were different among the three patient groups and between the CP and controls. Length of stay was significantly higher in the CN group compared to the controls, but none of the other clinical outcomes were significant. After adjusting the *p*-values for multiple comparisons, we found that certain cytokine and chemokine profiles were significantly associated with the clinical outcomes: 42 cytokine/chemokines with length of stay, 2 cytokines (B lymphocyte chemoattractant-CXCL13 and Interleukin-2R) with NEC, and 1 cytokine (macrophage migration inhibitory factor) with ROP (Supplementary Tables S2 and S3).

## 4. Discussion

To our knowledge, this is the first report investigating culture-negative sepsis with regard to microbiome diversity and composition across stool, skin, and blood—including bacterial, fungal, and viral components—as well as serum cytokine/chemokine biomarker profiles. We report significant differences in the stool and skin microbiome composition in preterm infants with culture-negative sepsis compared to controls, but no significant differences in cytokine or chemokine biomarker profiles. Notably, culture-negative infants had longer hospital stays than controls. These findings point to subtle but potentially meaningful microbiological shifts in the absence of systemic inflammatory markers.

While the alpha and beta diversity analyses of the microbiome did not reveal statistically significant differences among the three groups, the MaAsLin analysis identified differential relative abundances of specific bacterial and fungal genera in both the CN and CP groups compared to controls. In the stool microbiome, bacterial genera, including *Enterobacter*, *Streptococcus*, *Corynebacterium*, and *Enterococcus*, and fungal genera such as *Candida*, *Nakaseomyces*, and *Saccharomyces* were significantly altered between the CN and CO groups. Some of these genera were also found to be differentially abundant in the CP group, suggesting a potential shared microbial disturbance in both CN and CP sepsis phenotypes. However, the clinical relevance of these specific shifts in microbial composition remains unclear.

A growing body of evidence links early-life dysbiosis to neonatal complications, including sepsis [36,37]. Our findings support this, showing distinct differences in microbial composition between both CP and CN infants compared to controls. Using MaAsLin2, we identified taxa that were differentially abundant not only in the CP and CN infants compared to the CO, but also between the CP and CN groups themselves. This suggests that, while both sepsis groups show microbial disruption, the nature of that disruption may differ based on culture status. The CP infants demonstrated an inflammatory response and had identifiable pathogens in culture, whereas the CN infants showed overlapping but distinct microbial profiles without corresponding systemic cytokine elevations. This raises the possibility that in CN infants, dysbiosis may reflect a non-infectious inflammatory trigger or an early microbial imbalance not yet resulting in overt immune activation. The differences between CP and CN microbiomes further suggest that culture status may reflect biologically distinct processes rather than simply a difference in diagnostic yield.

Interestingly, despite the microbial differences between the CN/CP and controls, systemic immune activation was only observed in the CP group. The CP infants had elevated levels of several pro-inflammatory cytokines compared to both the CN and CO groups, confirming the presence of systemic inflammation during confirmed infection. In contrast, the CN infants had cytokine profiles that were indistinguishable from controls, suggesting that their clinical signs of sepsis were not driven by a measurable systemic inflammatory response. This refutes our original hypothesis and points to the possibility that non-infectious processes or localized inflammation may have triggered sepsis evaluations in the CN group. Our findings suggest that incorporating microbial community-level data and functional analyses (e.g., metabolomics, multi-omics) may be key to understanding the pathophysiology of both CN and CP sepsis [38,39].

The virome analysis showed low viral abundance overall, which may be attributed to several factors, including the expected postnatal development of the infant virome, limited enteral and environmental exposure in preterm infants, specific characteristics of our cohort, and technical aspects of sample collection, storage, and the virome assessment methods used in this study. During early life, the intestinal virome is predominantly acquired postnatally, as microscopically detectable virus-like particles are absent in the initial fecal samples (meconium) of newborns [8]. Within a week of birth, the number of virus-like particles in feces reaches 10^8^ per gram, originating from dietary, maternal, and/or environmental sources. In contrast, preterm infants, with limited exposure to enteral feeds and environmental interactions, likely experience a more constrained development of their virome, which may explain the lower viral composition and abundance in our samples. This underscores the importance of considering the developmental timeline when investigating the virome, suggesting that future studies should explore the temporal dynamics of viral colonization and persistence in early life.

Despite the overall low viral abundance, skin swab samples showed relatively higher viral detection, particularly in the CP group. This aligns with prior reports showing that the skin virome in neonates is often dominated by bacteriophages, papillomaviruses, polyomaviruses, and herpesviruses [40,41]. The presence of viral reads in a higher proportion of CP patients may reflect viral reactivation or co-infection during systemic illness. It also raises the possibility that the skin may serve as a viral reservoir during active sepsis. These findings should be interpreted with caution, however, given the methodological limitations inherent to virome studies in this population. Analyzing the virome in preterm infants presents additional technical challenges due to low viral loads, potential contamination, and the need for highly sensitive detection methods and advanced bioinformatics tools to accurately identify and interpret viral sequences.

Beyond biological factors, the NICU environment likely plays a significant role in shaping the skin microbiome and virome of preterm infants. These infants are frequently colonized by bacteria such as *Streptococcus*, *Staphylococcus*, *Neisseria*, and *Enterobacteriaceae*, which are organisms commonly found on the surfaces of stethoscopes, ventilators, incubators, and other equipment that comes into direct contact with their skin [18]. To reduce infections, preterm infants often receive topical antiseptics such as chlorhexidine, aimed at preventing central line-associated bloodstream infections and controlling resistant bacteria such as methicillin-resistant *S. aureus* [19]. However, the overall impact of the bacterial and fungal microbiome, along with that of the virome, remains unclear. It is likely that the complex interactions among these microbial communities influence which pathogens colonize the skin and how vulnerable infants are to sepsis [42,43].

While the unadjusted comparisons suggested differences in patient outcomes, these did not remain significant after accounting for prematurity and birth weight. The infants in the CN group initially appeared to have both a longer hospital stay and a higher incidence of BPD compared to controls, despite the absence of culture-confirmed infection or measurable systemic inflammation. However, these differences may be largely explained by the lower gestational age and birth weight observed in the CN group. In the multivariable regression analyses adjusting for these factors, neither the odds of BPD (*p* = 0.313) nor the length of stay (*p* = 0.065) were significantly different between the CN and CO groups. Similarly, the CP group no longer had significantly higher odds of BPD (adjusted *p* = 0.061) or ROP (adjusted *p* = 0.199) compared to controls. These findings support the interpretation that prematurity, rather than sepsis alone, accounts for the observed differences in outcomes. It remains possible that subtle metabolic, respiratory, or gastrointestinal issues—undetectable by serum cytokine assays—contributed to the clinical concern for sepsis in the CN group.

Limitations and Future Directions: Our pilot study has several limitations. First, the relatively small sample size limits both the generalizability of our findings and the ability to detect subtle differences between groups. We were unable to adjust for several potential confounding factors known to influence the neonatal microbiome, such as prior antibiotic exposure or feeding type, among others. Additionally, this study captured microbiome and cytokine/chemokine profiles at a single time point, which limits our ability to assess how these profiles evolve over time or how they relate to disease progression or resolution. Additionally, the delayed collection of stool samples (ranging from 0–17 days after sepsis evaluation) introduces potential variability, as the microbiome may have already been altered by the time of sample collection, compared to its state at the onset of clinical symptoms. There are several challenges in virome research, including difficulties with low biomass samples, confounding by host DNA, a lack of robust and curated virome databases, and contamination concerns. Additional limitations include the lack of a standardized definition or criteria for sepsis evaluation, which relied on the clinical judgment of the primary team. As a result, there is a potential for misclassification and overdiagnosis in the culture-negative sepsis group, since some infants may have exhibited symptoms due to non-infectious causes rather than true infection. Larger, long-term studies that look at both how microbes function (microbial metabolites) and how the immune system responds are needed to better understand the role of microbes and inflammation in neonatal sepsis, especially in the unclear but important group of infants with culture-negative sepsis.

## 5. Conclusions

In conclusion, this novel pilot study provides new insights into microbiome and inflammatory marker profiles in preterm neonates with suspected sepsis. As expected, the culture-positive group showed significantly elevated inflammatory markers. In contrast, the culture-negative group did not differ from controls in systemic inflammation or overall microbial diversity, though distinct microbial community shifts were observed in stool and skin samples. These findings suggest that even in the absence of inflammation, microbial differences may still influence immune function through local or subtle pathways. The limited viral detection likely reflects both technical challenges and the dynamic nature of the neonatal virome. Our results support antibiotic de-escalation in culture-negative cases when lab findings do not suggest infection. Future studies should explore whether these microbial patterns have diagnostic or prognostic value, and assess temporal dynamics and functional implications using longitudinal designs.

## Supplementary Material

supplementary file

**Supplementary Materials:** The following supporting information can be downloaded at: https://www.mdpi.com/article/10.3390/applmicrobiol5030057/s1, Figure S1: Microbiome of all samples in neonatal sepsis, Figure S2: Stool microbiome evaluation in the 3 groups, Figure S3: Skin microbiome evaluation in the 3 groups, Figure S4: Blood microbiome evaluation in the 3 groups, Figure S5: Stool mycobiome ITS2 evaluation in the 3 groups, Figure S6: Skin mycobiome ITS2 evaluation in the 3 groups, Figure S7: Skin Virome analyses, Table S1: Viruses identified in the skin, Table S2: Cytokine/Chemokine association with ‘length of stay outcome’, Table S3: Cytokine/Chemokine association with NEC, ROP and IVH & PVL, Table S4: List of organisms identified from the blood of preterm infants with culture-positive sepsis.

## Figures and Tables

**Table 1.** Baseline patient characteristics.

Characteristic	Group			*p*-Value
	CN (*n* = 21)	CO (*n* = 10)	CP (*n* = 19)	
Birth weight (g) ^2^	900 (690, 1140)	1250 (1040, 1620)	860 (665, 1250)	0.028 *
Gestational age (weeks) ^2^	27.1 (24.1, 29.0)	30.5 (27.9, 31.3)	25.9 (24.4, 28.1)	0.026 *
Male sex ^1^	8 (38.1)	4 (40.0)	12 (63.2)	0.287
Race ^1^				0.064
Asian	0 (0.0)	0 0.00	1 5.26	
Black	6 (28.6)	2 20.00	6 31.58	
Hispanic	9 (42.9)	0 (0.0)	4 (21.1)	
White	6 (28.6)	8 (80.0)	8 (42.1)	
Vaginal delivery^1^	9 (42.9)	0 (0.0)	6 (31.6)	0.048 *
Multiple births ^1^	5 (23.8)	5 (50.0)	2 (10.5)	0.058
Prenatal steroids ^1^	17 (81.0)	10 (100.0)	16 (84.2)	0.442
Antibiotics at birth ^1^	15 (71.4)	5 (50.0)	13 (68.4)	0.507
Apgar score at 1 min ^2^	7.0 (4.0, 7.0)	8.0 (7.0, 8.0)	4.0 (3.0, 7.0)	0.094
Apgar score at 5 min ^2^	8.0 (6.0, 8.0)	9.0 (8.0, 9.0)	7.0 (5.0, 9.0)	0.024 *
Age at sepsis evaluation (days) ^2^	20.0 (14.0, 29.0)	16.0 (12.0, 22.0)	37.0 (13.0, 52.0)	0.087

CN—culture-negative group; CO—control infants; CP—culture-positive group.

* Indicates statistically significant difference (α = 0.05).

1 Frequency (%), Fisher’s exact test *p*-value

2 median (inter-quartile range), Kruskal–Wallis test *p*-value

3 frequency (%), Kruskal–Wallis test *p*-value.

**Table 2.** MaAsLin analysis of microbiome and mycobiome differential abundance between the culture-negative and control groups.

CN vs. CO			Coef	Qval
**16S**	**Blood**	*Corynebacterium*	−1.929	0.282
		*Pseudomonas*	−1.106	0.282
		*Staphylococcus*	−1.766	0.282
		*Pelomonas*	−0.768	0.700
		*Family Burkholderiaceae*	−0.210	0.837
		*Geobacillus*	0.689	0.775
		*Thermus*	−0.930	0.775
		*Streptococcus*	−0.312	0.923
		*Ralstonia*	−0.090	0.934
		*Halomonas*	0.035	0.951
	**Skin**	*Staphylococcus*	2.514	0.009 *
		*Haemophilus*	2.092	0.128 *
		*Streptococcus*	1.486	0.226 *
		*Enterobacter*	1.330	0.513
		*Corynebacterium*	0.185	0.949
		*Pelomonas*	−0.051	0.949
		*Pseudomonas*	0.174	0.949
	**Stool**	*Enterobacter*	−0.496	0.000 *
		*Streptococcus*	4.875	0.002 *
		*Corynebacterium*	2.489	0.049 *
		*Enterococcus*	1.485	0.229 *
		*Family Clostridiaceae*	1.200	0.541
		*Staphylococcus*	0.485	0.541
**ITS2**	**Skin**	*Candida*	9.525	0.000 *
		*Saccharomyces*	−4.810	0.001 *
		*Malassezia*	0.847	0.458
	**Stool**	*Candida*	3.922	0.000 *
		*Nakaseomyces*	3.468	0.041 *
		*Saccharomyces*	3.016	0.052 *
		*Malassezia*	−0.204	0.919

CN—culture-negative group; CO—control infants. Coefficient (coef) represents the effect size of the association between the microbial feature and the outcome. A positive coef indicates a positive association, and a negative coef indicates a negative association, with the magnitude reflecting the strength of the relationship. Q value (Qval) refers to the false discovery rate (FDR)-adjusted *p*-value, calculated using the Benjamini–Hochberg procedure to account for multiple comparisons. A Qval ≤ 0.25 is considered statistically significant.

* Indicates statistically significant difference (α = 0.05).

**Table 3.** Comparison of cytokine/chemokine inflammatory biomarkers among the patient groups.

Cytokine/Chemokine	CN Median (IQR)	CO Median (IQR)	CP Median (IQR)	*p*-Value
APRIL	86.7 (44.6,275.4) ^A^	25.6 (10.5, 76.1) ^A^	416.6 (232.8,1266.4) ^B^	0.001 *
BAFF	37.4 (30.5, 44.1)	55.7 (48.9, 64.7)	41.7 (32.5, 48.4)	0.131
BLC-CXCL13	47.2 (41.6, 72.2) ^A^	35.5 (29.4, 55.6) ^A^	184.8 (84.7, 418.7) ^B^	0.001 *
CD30	918.2 (568.3, 1164.7)	587.0 (570.4, 981.1)	1102.4 (773.2, 1729.0)	0.120
CD40L	0.0 (0.0, 0.0) ^A^	0.0 (0.0, 11.8) ^A,B^	15.0 (3.7, 52.2) ^B^	0.009 *
ENA78-CXCL5	93.3 (79.1, 205.6)	218.6 (136.9, 252.4)	73.2 (46.6, 164.4)	0.114
Eotaxin-CCL11	7.0 (5.5, 8.3)	6.5 (6.0, 8.1)	11.5 (5.4, 16.7)	0.642
Eotaxin 2-CCL24	103.6 (91.5, 184.3)	126.1 (76.8, 142.8)	240.2 (205.4, 837.0)	0.046 *
Eotaxin 3-CCL26	0.1 (0.1, 0.2)	0.1 (0.1, 0.2)	0.2 (0.2, 0.3)	0.058
FGF 2	0.0 (0.0, 0.0)	0.0 (0.0, 5.1)	0.0 (0.0, 1.8)	0.533
Fractalkine-CX3CL1	0.0 (0.0, 0.0) ^A^	0.0 (0.0, 0.0) ^A^	4.1 (0.0, 6.2) ^B^	0.003 *
G-CSF	11.5 (6.2, 14.0) ^A^	11.5 (10.1, 14.0) ^A,B^	38.7 (20.9, 220.5) ^B^	0.017 *
GM-CSF	0.0 (0.0, 0.0) ^A,B^	0.0 (0.0, 0.0) ^A^	4.3 (0.0, 25.2) ^B^	0.015 *
GRO-alpha-CXCL1	6.5 (5.8, 9.0)	7.1 (5.5, 21.0)	13.5 (6.7, 34.1)	0.210
HGF	134.7 (77.5, 211.8) ^A,B^	68.3 (48.1, 69.1) ^A^	426.5 (176.2, 495.7) ^B^	0.002 *
ITAC-CXCL11	3.9 (3.3, 10.4)	3.3 (2.8, 9.3)	11.4 (4.8, 21.1)	0.051
IFN-alpha	0.0 (0.0, 0.0) ^A^	0.0 (0.0, 0.0) ^A^	0.9 (0.0, 1.8) ^B^	0.001 *
IFN-gamma	0.0 (0.0, 0.8) ^A^	0.0 (0.0, 0.0) ^A^	4.7 (2.4, 9.5) ^B^	0.007 *
IL1-alpha	0.0 (0.0, 0.0)	0.0 (0.0, 0.0)	4.2 (0.0, 5.5)	0.076
IL-1-beta	0.0 (0.0, 0.0) ^A^	0.0 (0.0, 0.0) ^A^	6.2 (1.7, 14.4) ^B^	0.005 *
IL-10	0.3 (0.0, 2.2) ^A,B^	0.0 (0.0, 0.7) ^A^	4.4 (2.6, 8.7) ^B^	0.016 *
IL-12p70	1.6 (1.4,1.9) ^A,B^	1.7 (1.3, 1.9) ^A^	2.6 (1.9, 3.6) ^B^	0.014 *
IL-13	0.0 (0.0, 0.0)	0.0 (0.0, 0.0)	0.0 (0.0, 2.7)	0.181
IL-15	5.8 (4.7, 10.2)	9.8 (8.7, 12.4)	8.8 (5.1, 14.4)	0.454
IL-16	313.3 (216.8, 479.9) ^A^	194.8 (129.6, 298.0) ^A^	720.1 (644.7, 910.8) ^B^	0.005 *
IL-17A-CTLA 8	0.3 (0.0, 0.9) ^A^	0.5 (0.0, 3.8) ^A^	3.2 (1.9, 6.3) ^B^	0.036 *
IL-18	37.1 (22.3, 49.6) ^A^	25.5 (12.7, 29.9) ^A^	81.2 (61.2, 189.3) ^B^	0.002 *
IL-2	2.2 (0.0, 4.9)	1.5 (0.0, 5.3)	11.0 (3.3, 18.6)	0.098
IL-20	2.8 (2.1, 4.1)	2.0 (1.4, 6.4)	9.2 (6.1, 16.3)	0.038 *
IL-21	0.0 (0.0, 5.5) ^A^	0.0 (0.0, 0.0) ^A^	12.3 (1.8, 27.0) ^B^	0.007 *
IL-22	0.0 (0.0, 15.6)	0.0 (0.0, 0.0)	26.8 (0.0, 48.9)	0.045 *
IL-23	0.0 (0.0, 6.8) ^A,B^	0.0 (0.0, 0.0) ^A^	10.4 (0.0, 42.9) ^B^	0.040 *
IL-27	0.0 (0.0, 0.0)	0.0 (0.0, 0.0)	0.0 (0.0, 0.0)	0.400
IL-2R	11548.5 (9430.1, 20,497.2) ^A,B^	9131.5 (7430.5, 11,656.5) ^A^	18153.4 (12,519.6, 38,817.7) ^B^	0.022 *
IL-3	0.0 (0.0, 0.0)	0.0 (0.0, 0.0)	0.0 (0.0, 12.3)	0.116
IL-31	0.0 (0.0, 0.0) ^A^	0.0 (0.0, 0.0) ^A^	1.1 (0.0, 23.7) ^B^	0.003 *
IL-4	6.0 (2.0, 11.3) ^A,B^	2.4 (1.0, 3.4) ^A^	17.8 (7.4, 26.5) ^B^	0.010
IL-5	0.0 (0.0, 0.0) ^A^	0.0 (0.0, 0.8) ^A^	1.9 (1.0, 4.5) ^B^	0.000 *
IL-6	0.0 (0.0, 7.9) ^A^	0.0 (0.0, 0.0) ^A^	43.6 (6.5, 132.0) ^B^	0.005 *
IL-7	0.5 (0.5, 0.6) ^A^	0.6 (0.4, 0.6) ^A,B^	0.9 (0.8, 1.3) ^B^	0.024 *
IL-8-CXCL8	9.7 (5.6,42.0) ^A,B^	5.3 (3.4, 11.5) ^A^	48.7 (19.8, 106.0) ^B^	0.007 *
IL-9	0.4 (0.0, 2.7) ^A^	0.0 (0.0, 3.6) ^A^	6.5 (4.1, 14.1) ^A,B^	0.013 *
IP-10-CXCL10	13.6 (7.9, 19.8)	7.8 (6.5, 10.0)	15.2 (11.7, 124.2)	0.054
LIF-15	3.2 (2.9, 3.4) ^A,B^	2.7 (2.5,4.1) ^A^	4.8 (3.9, 9.0) ^B^	0.029 *
M-CSF	3.2 (1.6, 9.3) ^A,B^	0.0 (0.0, 0.8) ^A^	12.4 (9.3, 41.4) ^B^	0.008 *
MCP-1-CCL2	140.3 (123.6, 206.6)	132.0 (93.0, 173.2)	327.4 (208.4, 585.5)	0.124
MCP-2-CCL8	3.0 (2.1, 5.0)	2.6 (2.4, 7.0)	4.1 (3.2, 18.6)	0.108
MCP-3-CCL7	0.0 (0.0, 1.4)	0.0 (0.0, 2.9)	2.5 (0.0, 11.5)	0.104
MDC	284.1 (214.1, 409.0)	257.3 (241.3, 343.2)	318.0 (263.1, 351.7)	0.875
MIF	13.0 (7.8, 20.4) ^A^	5.1 (4.8, 6.8) ^A^	57.3 (18.6, 110.9) ^B^	0.002 *
MIG-CXCL9	0.0 (0.0, 0.0) ^A^	0.0 (0.0, 0.0) ^A,B^	17.6 (0.0, 73.6) ^B^	0.007 *
MIP-1alpha-CCL3	5.1 (3.2, 7.7) ^A^	3.0 (2.6, 12.1) ^A,B^	10.9 (7.6, 19.8) ^B^	0.025 *
MIP-1beta-CCL4	36.9 (18.6, 45.1)	22.2 (14.6, 68.3)	73.2 (41.4, 126.1)	0.099
MIP-3alpha-CCL20	50.0 (29.9, 154.9)	20.4 (15.5, 24.1)	75.0 (38.8, 249.9)	0.047 *
MMP-1	10.3 (8.7, 17.1)	8.3 (2.2, 24.1)	16.1 (11.8, 19.4)	0.374
NGF-beta	0.4 (0.0, 2.3) ^A^	0.0 (0.0, 0.5) ^A^	3.4 (2.6, 4.7) ^B^	0.004 *
SCF	31.9 (16.6, 48.3) ^A,B^	16.1 (12.9, 21.9) ^A^	70.2 (42.1, 91.0) ^B^	0.011
SDF-1-alpha	337.2 (179.5, 684.3) ^A^	171.9 (149.6, 195.4) ^A^	889.1 (578.6, 1311.5) ^B^	0.002 *
TNF-alpha	4.4 (2.9, 7.1) ^A,B^	3.8 (1.8, 5.0) ^A^	12.5 (6.3, 20.5) ^B^	0.030 *
TNF-beta	0.2 (0.0, 1.8)	0.5 (0.0, 1.7)	3.8 (1.7, 10.6)	0.031 *
TNF-RII	155.7 (113.1, 213.0) ^A^	80.1 (77.6, 101.8) ^A^	273.2 (193.9, 340.2) ^B^	<0.001 *
TRAIL	0.0 (0.0, 6.4) ^A^	0.0 (0.0, 0.0) ^A^	11.9 (1.3, 21.8) ^B^	0.012 *
TSLP	2.1 (1.6, 2.5) ^A,B^	1.9 (1.5, 2.1) ^A^	3.2 (2.2, 3.9) ^B^	0.017 *
TWEAK	169.9 (119.8, 191.5) ^A^	156.2 (113.5, 184.3) ^A,B^	323.9 (192.5, 445.4) ^B^	0.040 *
VEGF-A	189.5 (109.2, 298.5) ^A,B^	81.7 (78.3, 294.1) ^A^	387.9 (290.6, 515.8) ^B^	0.022 *

* Significant *p*-value for the difference between the 3 groups.

A,B Groups with different superscript letters are significantly different from each other. Because the cytokine levels were not normally distributed, as determined by the Shapiro–Wilk test, the Kruskal–Wallis test was used to compare the three groups’ cytokine levels. For cytokines that differed significantly (α = 0.05) across the three groups, a post-hoc analysis was performed using Dunn’s test with Bonferroni correction for multiple comparisons in order to perform pairwise group comparisons. Note that sometimes there are no significant pairwise group differences despite a significant Kruskal–Wallis test because the post-hoc analysis is conservative. We found that the preterm infants with CP sepsis exhibited significantly elevated levels of 24 inflammatory markers compared to the CN sepsis group and 32 inflammatory markers compared to the asymptomatic healthy controls. We did not find significant differences in cytokines/chemokines between the CN and CO groups.

**Abbreviations:** APRIL—a proliferation-inducing ligand, BAFF—B-cell-activating factor, BLC—B lymphocyte chemoattractant, CXCL13—CXC motif chemokine ligand 13, CD—cluster differentiating antigen, CD40L—cluster differentiating antigen 40 ligand, ENA-78—epithelial-derived neutrophil-activating peptide 78, CCL—chemokine (C-C motif) ligand, FGF—fibroblast growth factor, Fractalkine-CX3CL1: chemokine (C-X3-C motif) ligand 1, G-CSF: granulocyte colony-stimulating factor, GM-CSF—granulocyte-macrophage colony-stimulating factor, Gro-alpha—growth-related oncogene-alpha, HGF—hepatocyte growth factor, ITAC—interferon-inducible T-cell alpha chemoattractant, IFN—interferon, IL—interleukin, IP—interferon gamma-induced protein, CTLA—cytotoxic T lymphocyte-associated antigen, LIF—leukemia inhibitory factor, M-CSF—macrophage colony-stimulating factor, MCP—monocyte chemoattractant protein, MDC—macrophage-derived chemokine, MIF—macrophage migration inhibitory factor, MIP— macrophage inflammatory protein, MMP—matrix metalloproteinase, NGF—nerve growth factor, SCF—stem cell factor, SDF—stromal cell-derived factor, TNF—tumor necrosis factor, TNF-RII—tumor necrosis factor receptor 2, TRAIL—TNF-related apoptosis-inducing ligand, TSLP—thymic stromal lymphopoietin, TWEAK—tumor necrosis factor-like weak inducer of apoptosis, VEGF—vascular endothelial growth factor.

**Table 4.** Association of neonatal sepsis groups with outcomes.

Outcomes	Group			*p*-Value
	CN (*n* = 21)	CO (*n* = 10)	CP (*n* = 19)	
NEC^1^	3 (14.3)	0 (0.0)	5 (26.3)	0.190
Any ROP^1^	16 (76.2) ^A,B^	4 (40.0) ^A^	16 (84.2) ^B^	0.039 *
BPD ^1†^	16 (76.2) ^B^	3 (30.0) ^A^	17 (89.5) ^B^	0.002*
BPD severity ^3^	*n* = 16	*n* = 3	*n* = 17	0.070
Mild	4 (25.0)	2 (66.7)	1 (5.9)	
Moderate	3 (18.9)	1 (33.3)	3 (17.7)	
Severe	9 (56.3)	0 (0.0)	13 (76.5)	
Severe ROP or ROP surgery ^1^	2 (9.1)	0 (0.0)	4 (22.2)	0.229
Any IVH ^1^	10 (47.6)	1 (10.0)	5 (26.3)	0.087
Severe IVH or PVL ^1^	3 (14.3)	0 (0.0)	1 (5.3)	0.515
Length of stay (days) ^2^	119.0 ^A^ (76.0, 160.0)	50.5 ^B^ (46.0, 90.0)	166.0 ^C^ (144.0, 264.0)	<0.001 *

CN—culture-negative group; CO—control infants; CP—culture-positive group

* indicates statistically significant difference (α = 0.05).

A,B,C Groups with different superscript letters are significantly different from each other (groups sharing the same superscript letter are not) after Bonferroni adjustment for multiple comparisons.

1 Frequency (%), Fisher’s exact test *p*-value

2 median (inter-quartile range), Kruskal–Wallis test *p*-value

3 frequency (%), Kruskal–Wallis test *p*-value.

## Data Availability

The raw data supporting the conclusions of this article will be made available by the authors on request.

## Abbreviations

The following abbreviations are used in this manuscript:

16S: 16S ribosomal RNA (used for bacterial microbiome analysis)
ANOVA: Analysis of Variance
ATIMA: Agile Toolkit for Incisive Microbial Analyses
BCL: Binary base call
BPD: Bronchopulmonary dysplasia
cDNA: Complementary DNA
CP: Culture-positive sepsis
CMMR: Center for Metagenomics and Microbiome Research
CN: Culture-negative sepsis
CO: Control group (asymptomatic preterm neonates)
CXCL13: B lymphocyte chemoattractant
DNA: Deoxyribonucleic acid
ITS2: Internal transcribed spacer 2 (used for fungal microbiome analysis)
LOS: Length of stay
MaAsLin2: Microbiome Multivariable Association with Linear Models 2
NEC: Necrotizing enterocolitis
NGS: Next-generation sequencing
NICU: Neonatal Intensive Care Unit
ROP: Retinopathy of prematurity
RNA: Ribonucleic acid
SDI: Shannon Diversity Index
TSS: Total Sum Scaling
