# Supplementary material for: Microbiome Signatures and Inflammatory Biomarkers in Culture-Negative Neonatal Sepsis
Source: Appl Microbiol (Basel). Author manuscript; Available in PMC 2026 Mar 28. (PMC13022588; doi:10.3390/applmicrobiol5030057)
Supplement: supplementary file [file NIHMS2152835-supplement-supplementary_file.pdf]

SUPPLEMENTARY FILE

Table of contents

I. Supplementary Figure S1: Microbiome of all samples in neonatal sepsis

II. Supplementary figure S2: Stool microbiome evaluation in the 3 groups

III. Supplementary figure S3: Skin microbiome evaluation in the 3 groups

IV. Supplementary figure S4: Blood microbiome evaluation in the 3 groups

V. Supplementary figure S5: Stool mycobiome ITS2 evaluation in the 3 groups

VI. Supplementary figure S6: Skin mycobiome ITS2 evaluation in the 3 groups

VII. Supplementary figure S7: Skin Virome analyses

VIII. Supplementary table 1: Viruses identified in the skin

IX. Supplementary table 2: Cytokine/Chemokine association with 'length of stay outcome'

X. Supplementary table 3: Cytokine/Chemokine association with NEC, ROP and IVH & PVL

XI. Supplementary table 4: List of organisms identified from the blood of preterm infants with culture-positive sepsis

I. Supplementary Figure S1: Microbiome of all samples in neonatal sepsis

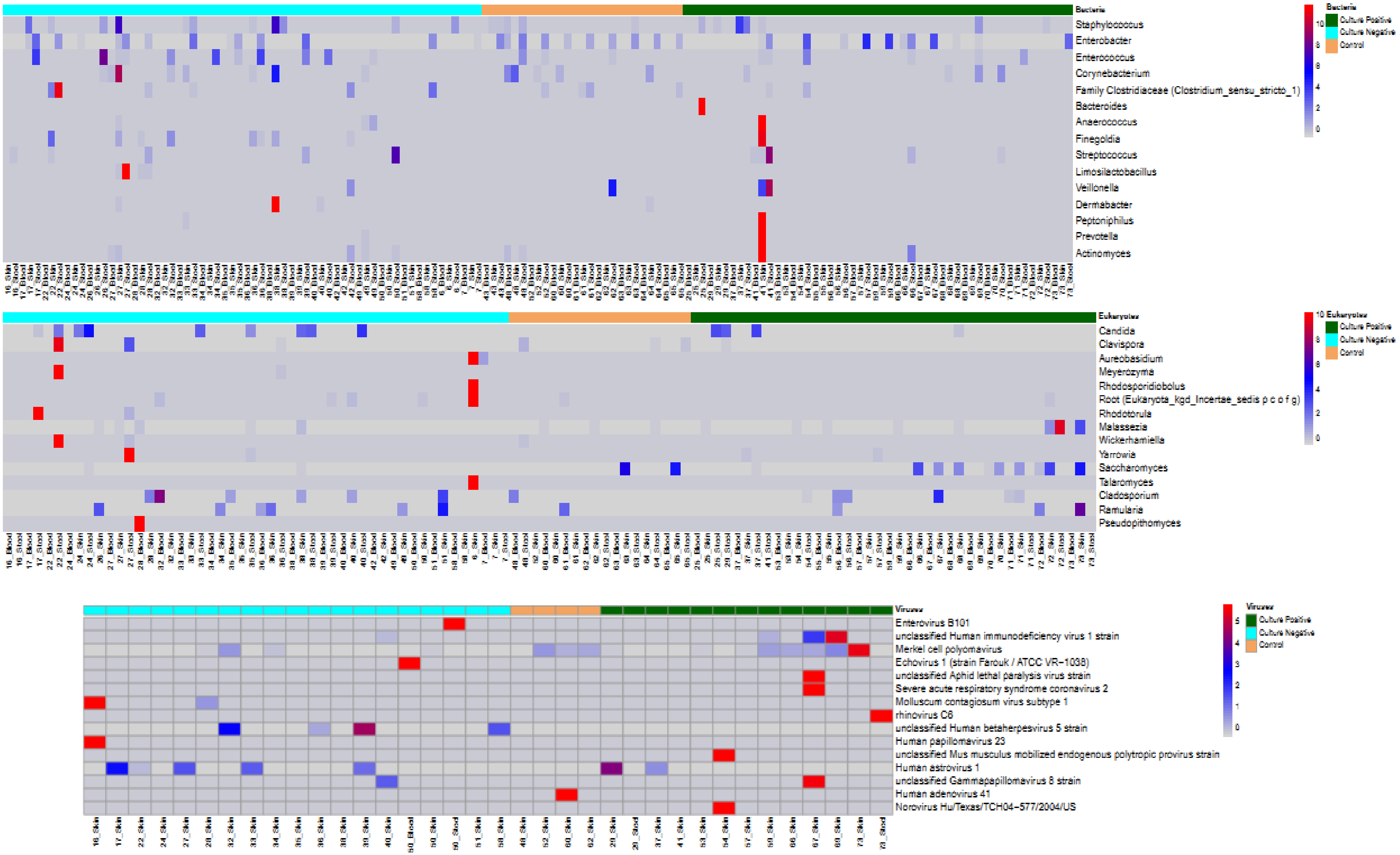

Figure S1 footnote: Microbiome composition across all samples is visualized in the heatmap, with bacterial, fungal, and viral taxa displayed for culture-negative (light blue), control (orange), and culture-positive (green) groups. While variations in individual taxa are observed, no distinct clustering or consistent differences are apparent between the three groups, suggesting an overall similarity in microbiome profiles regardless of culture status.

II. Supplementary figure S2: Stool microbiome evaluation in the 3 groups

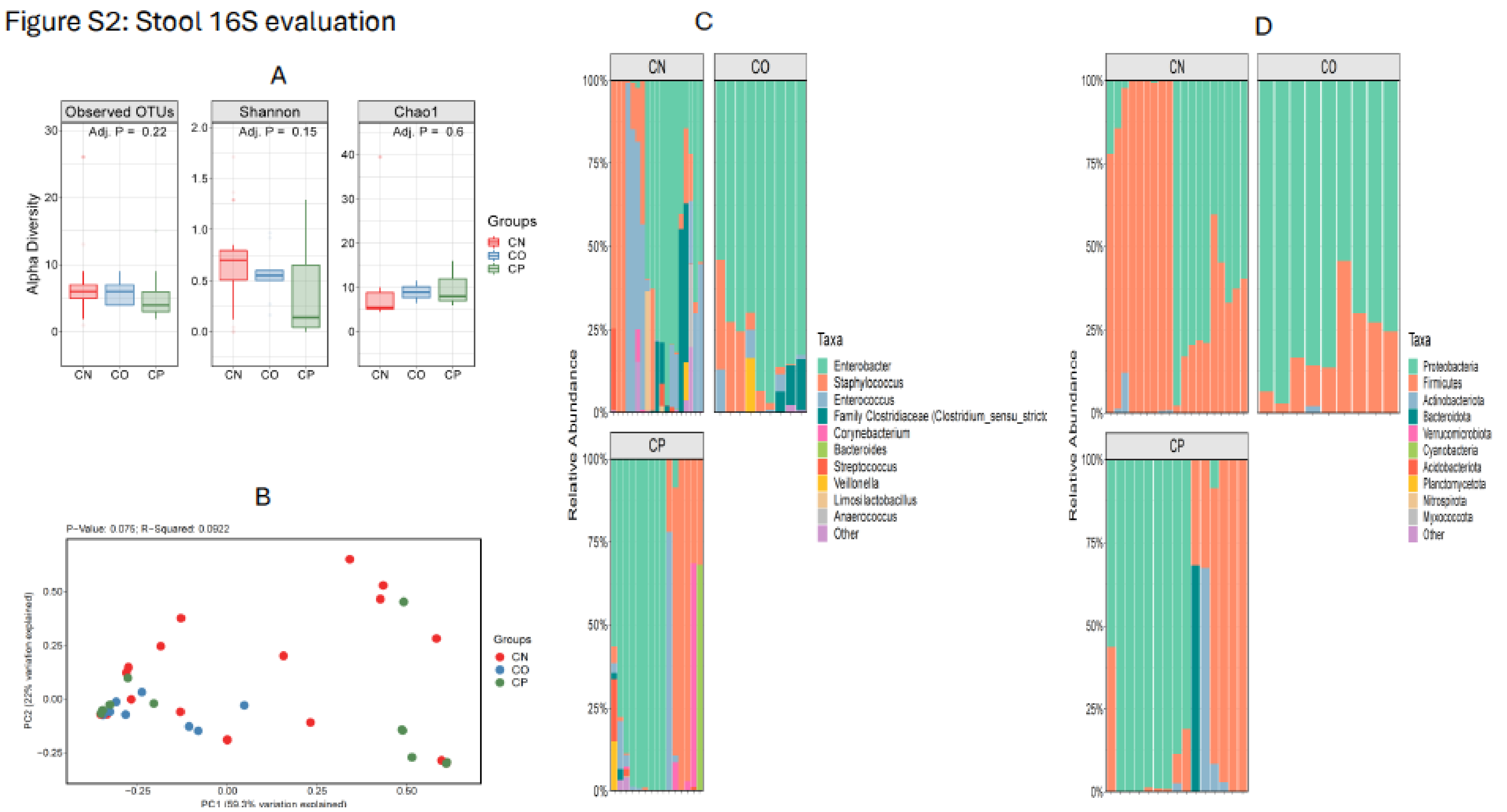

Figure S2 footnote: Stool microbiome analysis across culture-negative (CN), culture-positive (CP), and control (CO) groups. (A) Alpha diversity metrics (Observed OTUs, Shannon, and Chao1 indices) show no significant differences between groups, indicating similar within-sample microbial diversity. (B) Principal Coordinate Analysis (PCoA) based on beta diversity suggests no distinct clustering between groups ($P = 0.075$, $R^2 = 0.0522$), implying a lack of strong separation in microbial community composition. (C) Taxonomic relative abundance at the genus level shows a broadly similar microbial distribution across groups, with some variation in specific taxa. (D) Phylum-level composition reveals a predominance of Firmicutes and Proteobacteria across all groups, without distinct shifts between CN, CP, and CO samples. Overall, these findings indicate no significant differences in stool microbiome diversity or composition between the three groups.

III. **Supplementary figure S3: Skin microbiome evaluation in the 3 groups**

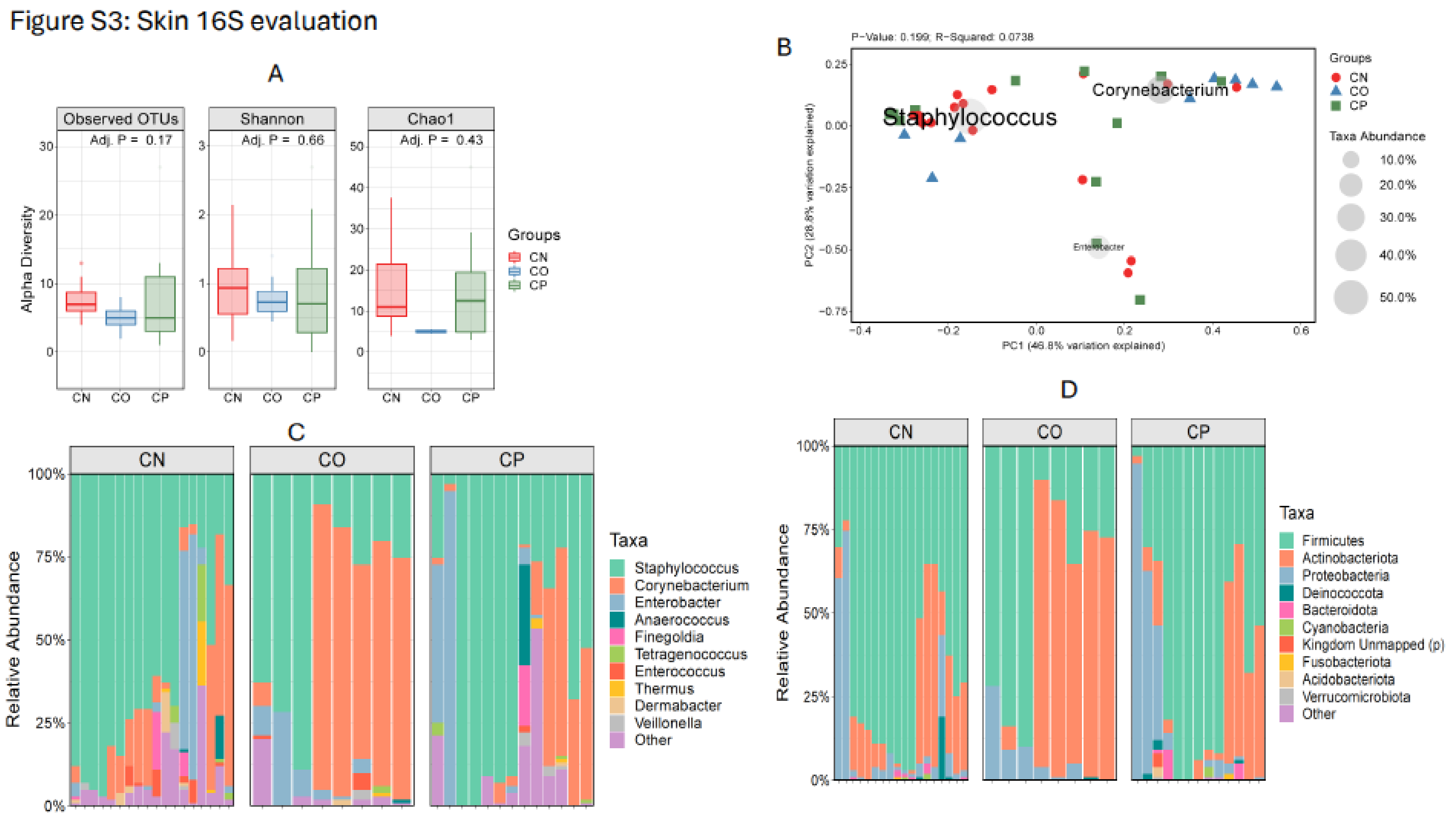

Figure S3 footnote: Skin microbiome analysis across culture-negative (CN), culture-positive (CP), and control (CO) groups. (A) Alpha diversity metrics (Observed OTUs, Shannon, and Chao1 indices) reveal no significant differences between groups, suggesting comparable within-sample microbial diversity. (B) Principal Coordinate Analysis (PCoA) demonstrates no clear clustering of microbial communities among groups ($P = 0.199$, $R^2 = 0.0738$), indicating a lack of strong differentiation in skin microbiome composition. (C) Genus-level relative abundance shows Staphylococcus and Corynebacterium as dominant taxa across all groups, with some variation in minor taxa. (D) Phylum-level composition highlights the predominance of Firmicutes and Actinobacteriota, with no striking differences between CN, CP, and CO groups. Overall, the skin microbiome composition appears largely similar among groups, without significant diversity or compositional shifts.

IV. Supplementary figure S4: Blood microbiome evaluation in the 3 groups

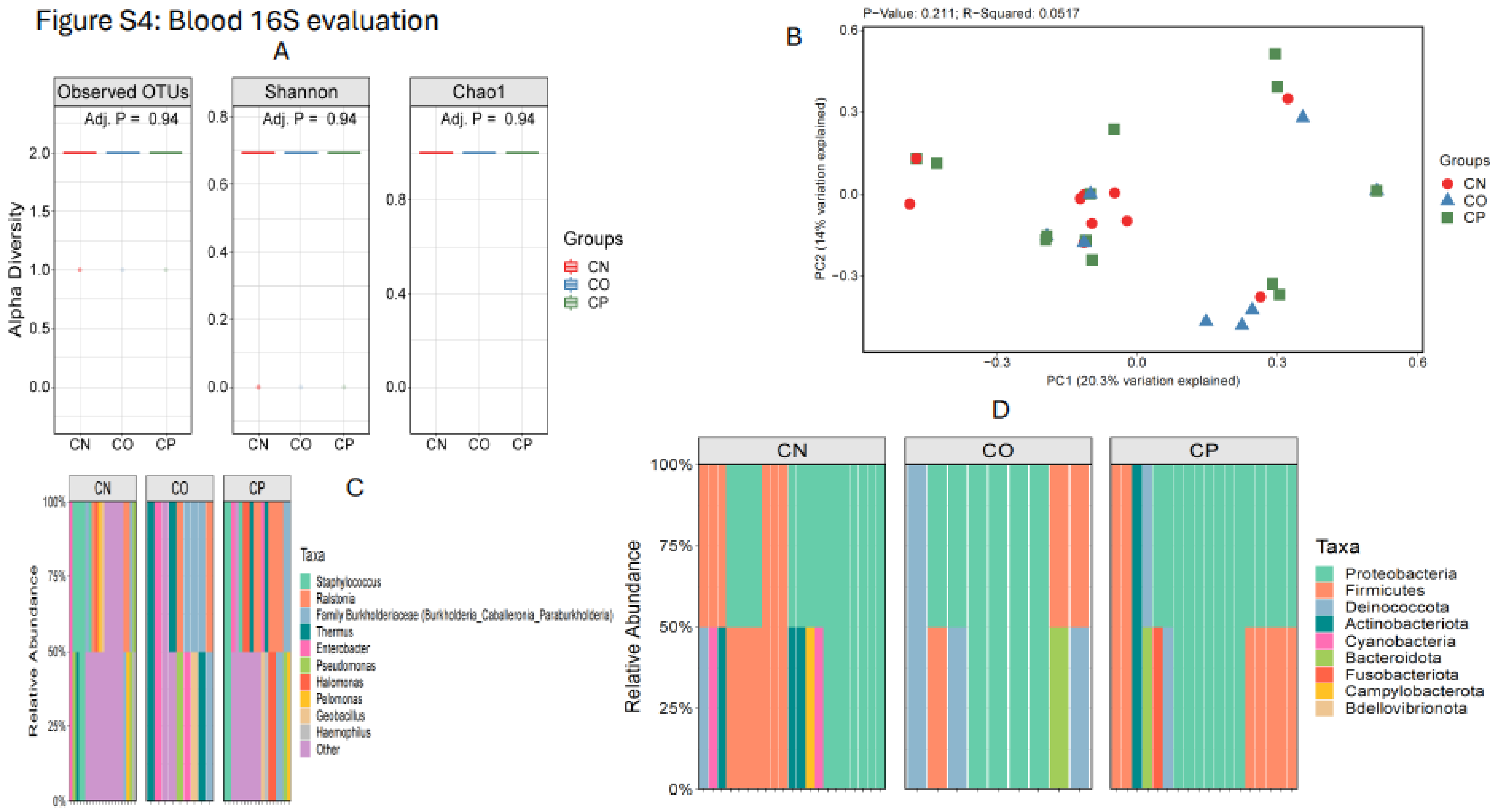

Figure S4 footnote: Blood microbiome analysis across culture-negative (CN), culture-positive (CP), and control (CO) groups. (A) Alpha diversity metrics (Observed OTUs, Shannon, and Chao1 indices) reveal no significant differences between groups (adjusted P = 0.94), suggesting comparable within-sample microbial diversity. (B) Principal Coordinate Analysis (PCoA) demonstrates no clear clustering of microbial communities among groups (P = 0.211, R^2 = 0.0517), indicating a lack of strong differentiation in blood microbiome composition. (C) Genus-level relative abundance shows *Staphylococcus*, *Ralstonia*, and *Pseudomonas* as dominant taxa across all groups, with variations in minor taxa. (D) Phylum-level composition highlights the predominance of Proteobacteria, Firmicutes, and Actinobacteriota, with an increased relative abundance of Proteobacteria in CP samples. Overall, the blood microbiome composition appears to vary across groups, but without significant diversity or compositional shifts

V. **Supplementary figure S5: Stool ITS2 mycobiome evaluation in the 3 groups**

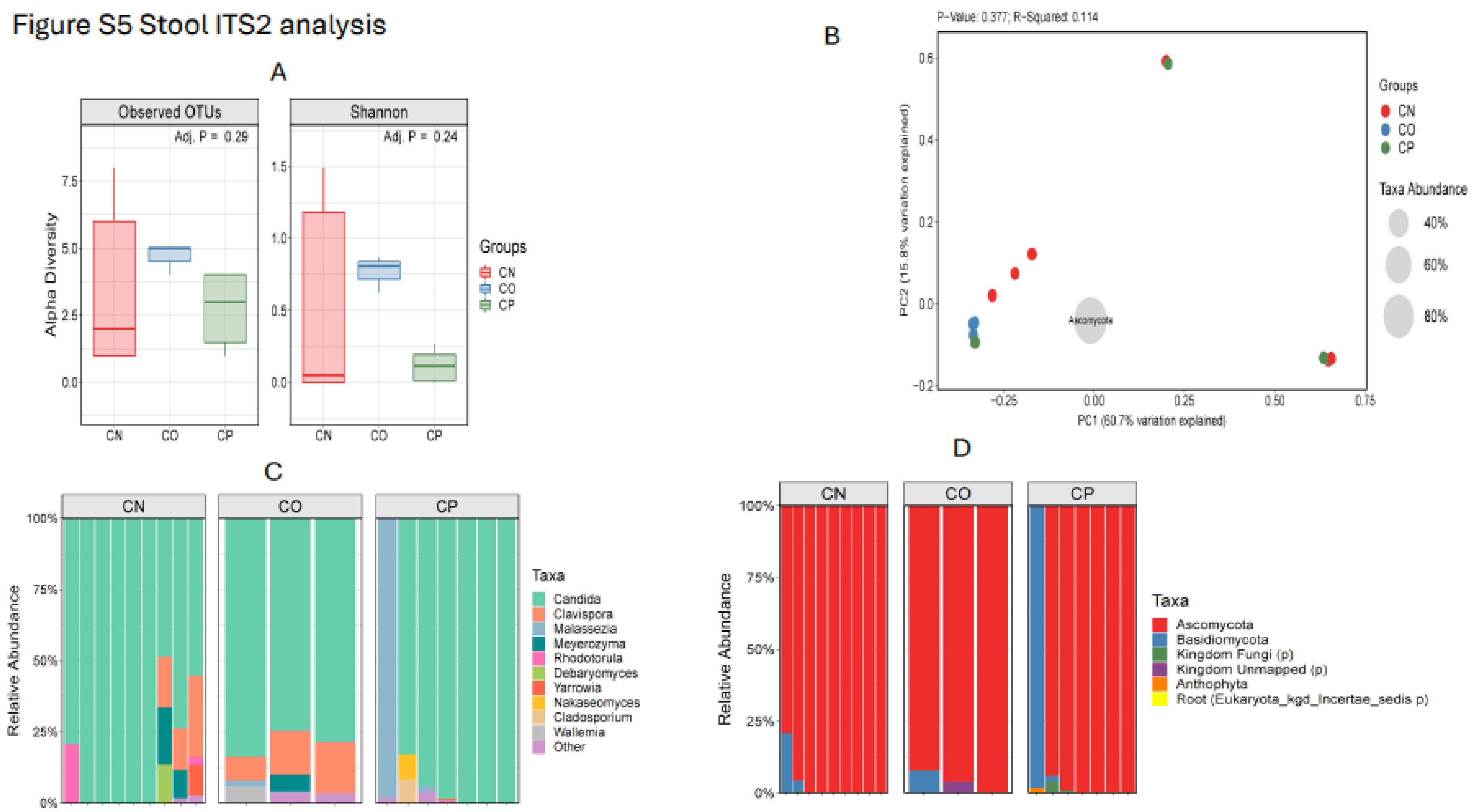

Figure S5 footnote: Stool mycobiome analysis across culture-negative (CN), culture-positive (CP), and control (CO) groups. (A) Alpha diversity metrics (Observed OTUs and Shannon indices) reveal no significant differences between groups (adjusted P = 0.29 and 0.24, respectively), suggesting comparable within-sample fungal diversity. (B) Principal Coordinate Analysis (PCoA) demonstrates no clear clustering of fungal communities among groups (P = 0.377, $R^2 = 0.114$), indicating a lack of strong differentiation in stool mycobiome composition. (C) Genus-level relative abundance shows *Candida*, *Clavispora*, and *Malassezia* as dominant taxa, with variation in minor fungal taxa across groups. (D) Phylum-level composition highlights the predominance of *Ascomycota* across all groups, with smaller contributions from *Basidiomycota* and other fungal taxa. Overall, the stool mycobiome composition varies across groups, but without significant diversity or compositional shifts.

VI. **Supplementary figure S6: Skin ITS2 mycobiome evaluation in the 3 groups**

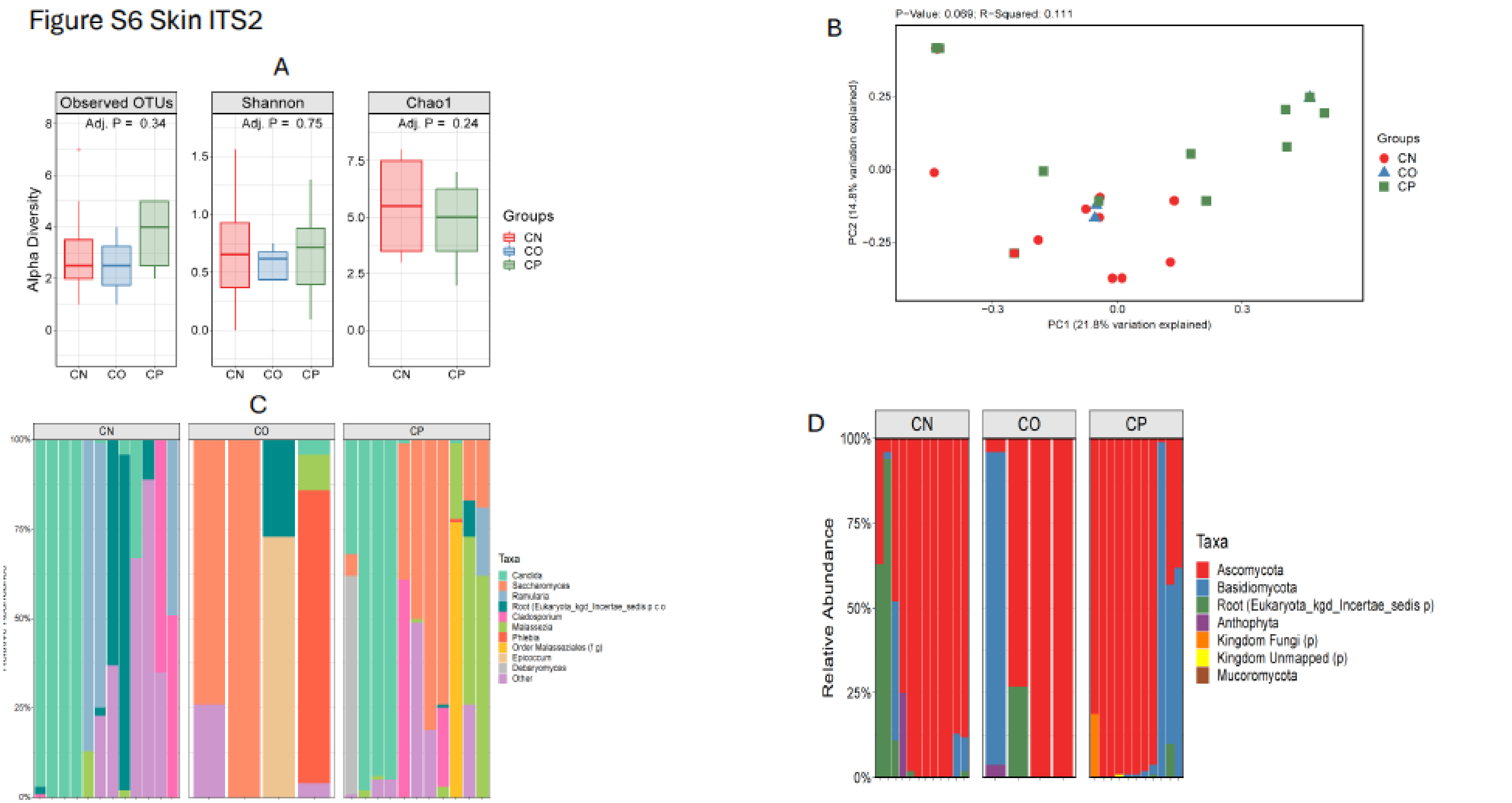

Figure S6 footnote: Skin mycobiome analysis across culture-negative (CN), culture-positive (CP), and control (CO) groups. (A) Alpha diversity metrics (Observed OTUs, Shannon, and Chao1 indices) show no significant differences between groups (adjusted P-values > 0.05), suggesting similar within-sample fungal diversity. (B) Principal Coordinate Analysis (PCoA) based on Bray-Curtis dissimilarity indicates no strong clustering among groups ($P = 0.098$, $R^2 = 0.111$), implying limited differentiation in fungal community composition. (C) Genus-level relative abundance highlights variation in fungal taxa distribution, with Candida, Malassezia, and Saccharomyces as predominant genera across groups. (D) Phylum-level composition reveals Ascomycota as the dominant fungal phylum, followed by Basidiomycota, with no major compositional shifts between CN, CP, and CO groups. Overall, the skin mycobiome appears relatively stable across all groups, with some variability in minor taxa.

VII. **Supplementary figure S7: Skin Virome evaluation in the 3 groups**

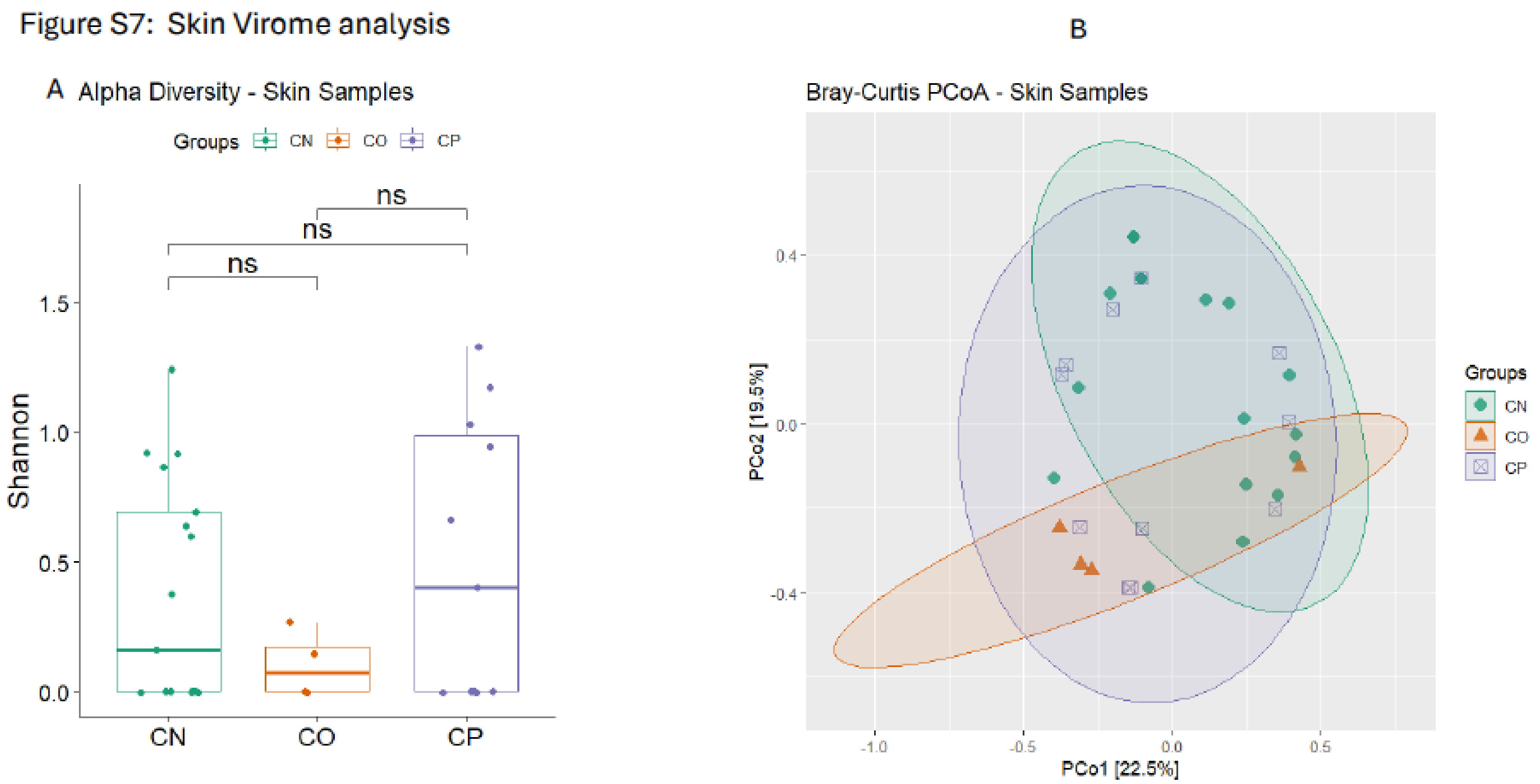

Figure S7 footnote: Skin virome analysis across culture-negative (CN), culture-positive (CP), and control (CO) groups. (A) Alpha diversity analysis (Shannon index) shows no significant differences between groups (ns), suggesting comparable viral diversity across skin samples. (B) Principal Coordinate Analysis (PCoA) based on Bray-Curtis dissimilarity reveals some separation between groups, with CO samples clustering more distinctly from CN and CP, though there is still substantial overlap. Overall, the skin virome composition exhibits some variation among groups, but without significant shifts in diversity.

VIII. Supplementary table 1: Viruses isolated from the skin

Viruses identified in the skin, 24 viruses across 32 skin samples

<table>
<tr><th>accession</th><th>lineage</th><th>sample_counts</th></tr>
<tr><td>HM011556.1</td><td>Viruses|Cossaviricota|Papovaviricetes|Sepolyvirales|Polyomaviridae|Alphapolyomavirus|Alphapolyomavirus quintihominis|Merkel cell polyomavirus|Merkel cell polyomavirus</td><td>25</td></tr>
<tr><td>Z25771.1</td><td>Viruses|Pisuviricota|Stelpaviricetes|Stellavirales|Astroviridae|Mamastrovirus|Mamastrovirus 1|Human astrovirus 1|Human astrovirus 1</td><td>7</td></tr>
<tr><td>KY490071.1</td><td>Viruses|Peploviricota|Herviviricetes|Herpesvirales|Herpesviridae|Cytomegalovirus|Human betaherpesvirus 5|unclassified Human betaherpesvirus 5 subspecies/strain|unclassified Human betaherpesvirus 5 strain</td><td>4</td></tr>
<tr><td>NC_001802.1</td><td>Viruses|Artverviricota|Revtraviricetes|Ortervirales|Retroviridae|Lentivirus|Human immunodeficiency virus 1|unclassified Human immunodeficiency virus 1 subspecies/strain|unclassified Human immunodeficiency virus 1 strain</td><td>4</td></tr>
<tr><td>FM955837.2</td><td>Viruses|Cossaviricota|Papovaviricetes|Zurhausenvirales|Papillomaviridae|Betapapillomavirus|Betapapillomavirus 1|Human papillomavirus 98|Human papillomavirus 98</td><td>2</td></tr>
<tr><td>KY040275.1</td><td>Viruses|Nucleocytoviricota|Pokkesviricetes|Chitovirales|Poxviridae|Molluscipoxvirus|Molluscum contagiosum virus|Molluscum contagiosum virus subtype 1|Molluscum contagiosum virus subtype 1</td><td>2</td></tr>
<tr><td>MF588704.1</td><td>Viruses|Cossaviricota|Papovaviricetes|Zurhausenvirales|Papillomaviridae|Gammapapillomavirus|Gammapapillomavirus 8|unclassified Gammapapillomavirus 8 subspecies/strain|unclassified Gammapapillomavirus 8 strain</td><td>2</td></tr>
<tr><td>MF588720.1</td><td>Viruses|Cossaviricota|Papovaviricetes|Zurhausenvirales|Papillomaviridae|Gammapapillomavirus|Gammapapillomavirus 12|unclassified Gammapapillomavirus 12 subspecies/strain|unclassified Gammapapillomavirus 12 strain</td><td>2</td></tr>
<tr><td>AB365435.1</td><td>Viruses|Pisuviricota|Pisoniviricetes|Picornavirales|Caliciviridae|Norovirus|Norwalk virus|Norovirus Hu/Texas/TCH04-577/2004/US|Norovirus Hu/Texas/TCH04-577/2004/US</td><td>1</td></tr>
<tr><td>AF536531.1</td><td>Viruses|Pisuviricota|Pisoniviricetes|Picornavirales|Dicistroviridae|Cripavirus|Aphid lethal paralysis virus|unclassified Aphid lethal paralysis virus subspecies/strain|unclassified Aphid lethal paralysis virus strain</td><td>1</td></tr>
<tr><td>D00530.1</td><td>Viruses|Pisuviricota|Pisoniviricetes|Sobelivirales|Solemoviridae|Polerovirus|Potato leafroll virus|unclassified Potato leafroll virus subspecies/strain|unclassified Potato leafroll virus strain</td><td>1</td></tr>
<tr><td>FJ544577.2</td><td>Viruses|Artverviricota|Revtraviricetes|Ortervirales|Retroviridae|Gammaretrovirus|Mus musculus mobilized endogenous polytropic provirus|unclassified Mus musculus mobilized endogenous polytropic provirus subspecies/strain|unclassified Mus musculus mobilized endogenous polytropic provirus strain</td><td>1</td></tr>
<tr><td>FM955841.1</td><td>Viruses|Cossaviricota|Papovaviricetes|Zurhausenvirales|Papillomaviridae|Betapapillomavirus|Betapapillomavirus 1|Human papillomavirus 105|Human papillomavirus 105</td><td>1</td></tr>
<tr><td>FR872816.1</td><td>Viruses|Artverviricota|Revtraviricetes|Ortervirales|Retroviridae|Gammaretrovirus|Murine leukemia-related retroviruses|Xenotropic MuLV-related virus|Xenotropic MuLV-related virus</td><td>1</td></tr>
<tr><td>GQ225794.1</td><td>Viruses|Duplornaviricota|Resentoviricetes|Reovirales|Sedoreoviridae|Rotavirus|Rotavirus A|Rotavirus A AU32xUK reassortant (UKg9AU32)|Rotavirus A AU32xUK reassortant (UKg9AU32)</td><td>1</td></tr>
<tr><td>JX413109.1</td><td>Viruses|Cossaviricota|Papovaviricetes|Zurhausenvirales|Papillomaviridae|Gammapapillomavirus|Gammapapillomavirus 19|Human papillomavirus type 161|Human papillomavirus type 161</td><td>1</td></tr>
<tr><td>KX514417.1</td><td>Viruses|Cossaviricota|Papovaviricetes|Zurhausenvirales|Papillomaviridae|Alphapapillomavirus|Alphapapillomavirus 7|human papillomavirus 39|human papillomavirus 39</td><td>1</td></tr>
<tr><td>KY316160.1</td><td>Viruses|Preplasmiviricota|Tectiliviricetes|Rowavirales|Adenoviridae|Mastadenovirus|Human mastadenovirus F|Human adenovirus 41|Human adenovirus 41</td><td>1</td></tr>
<tr><td>MF588699.1</td><td>Viruses|Cossaviricota|Papovaviricetes|Zurhausenvirales|Papillomaviridae|Gammapapillomavirus|Gammapapillomavirus 7|unclassified Gammapapillomavirus 7 subspecies/strain|unclassified Gammapapillomavirus 7 strain</td><td>1</td></tr>
<tr><td>NC_001716.2</td><td>Viruses|Peploviricota|Herviviricetes|Herpesvirales|Herpesviridae|Roseolovirus|Human betaherpesvirus 7|unclassified Human betaherpesvirus 7 subspecies/strain|unclassified Human betaherpesvirus 7 strain</td><td>1</td></tr>
<tr><td>NC_045512.2</td><td>Viruses|Pisuviricota|Pisoniviricetes|Nidovirales|Coronaviridae|Betacoronavirus|Severe acute respiratory syndrome-related coronavirus|Severe acute respiratory syndrome coronavirus 2|Severe acute respiratory syndrome coronavirus 2</td><td>1</td></tr>
<tr><td>U31779.1</td><td>Viruses|Cossaviricota|Papovaviricetes|Zurhausenvirales|Papillomaviridae|Betapapillomavirus|Betapapillomavirus 1|human papillomavirus 21|human papillomavirus 21</td><td>1</td></tr>
<tr><td>U31781.1</td><td>Viruses|Cossaviricota|Papovaviricetes|Zurhausenvirales|Papillomaviridae|Betapapillomavirus|Betapapillomavirus 2|Human papillomavirus 23|Human papillomavirus 23</td><td>1</td></tr>
<tr><td>X74462.1</td><td>Viruses|Cossaviricota|Papovaviricetes|Zurhausenvirales|Papillomaviridae|Alphapapillomavirus|Alphapapillomavirus 2|Human papillomavirus 3|Human papillomavirus 3</td><td>1</td></tr>
</table>

IX. Supplementary table -2: Correlations of cytokine/chemokine biomarkers with 'Length of stay'.

	Cytokine/chemokine	Correlation (r)	BH p-value
1	BAFF	0.4827453667	0.021429
2	CD30	0.4716049544	0.025994
3	CD40L	0.5281972037	0.013967
4	Eotaxin-2 (CCL24)	0.4934191531	0.017819
5	Fractalkine (CX3CL1)	0.4721171629	0.025994
6	GM-CSF	0.5302943091	0.014343
7	IFN-alpha	0.5011522798	0.015494
8	IFN-gamma	0.5042420698	0.015323
9	IL-1-alpha	0.538677035	0.019969
10	IL-1-beta	0.5039228058	0.015323
11	IL-10	0.5186442066	0.013967
12	IL-12p70	0.5063567427	0.015323
13	IL-13	0.5186442066	0.015323
14	IL-15	0.5948220601	0.015323
15	IL-17A (CTLA-8)	0.4938453304	0.01142
16	IL-18	0.5068571385	0.007995
17	IL-2	0.5609715447	0.015323
18	IL-20	0.5058350869	0.01142
19	IL-21	0.5267362427	0.015323
20	IL-22	0.6067865347	0.013967
21	IL-23	0.5432731678	0.006994
22	IL-27	0.5115211037	0.013967
23	IL-3	0.5290191655	0.025994
24	IL-31	0.4736402039	0.013967
25	IL-4	0.5026306381	0.015365
26	IL-5	0.5344117459	0.013967
27	IL-7	0.5120449311	0.015323
28	IL-9	0.6092133759	0.006994
29	LIF-15	0.5296598526	0.013967
30	M-CSF	0.5565909248	0.01142
31	MCP-3 (CCL7)	0.5785957862	0.009212
32	MIP-1-alpha (CCL3)	0.5732001472	0.017819
33	MMP-1	0.5565909248	0.009411

34	NGF-beta	0.5096599624	0.015323
35	SCF-39	0.5853129893	0.008846
36	SDF-1alpha	0.5408340456	0.013967
37	TNF-alpha	0.5535503421	0.015323
38	TNF-beta	0.5134810631	0.015323
39	TNF-RII	0.5103603816	0.01142
40	TRAIL-58	0.4843665332	0.021139
41	TSLP-80	0.6345167549	0.006994
42	TWEAK	0.5099106115	0.015323

X. Supplementary table 3: Cytokine/Chemokine associations with NEC, ROP and IVH & PVL

Cytokine/chemokine	Outcome	Odds Ratio	Lower CI	Upper CI	BH p-value
BLC-CXCL13	NEC	1.00549	1.000616	1.011438	0.037848
IL-2R	NEC	1.000048	1.000006	1.000105	0.045683
MIF	ROP or ROP surgery	1.015211	1.000416	1.032366	0.046263

Supplementary table 3 footnote: BH- Benjamini Hochberg correction for multiple comparisons, CI – confidence interval. Predictors are the list of cytokine/chemokine biomarker. LOS- length of stay, NEC- necrotizing enterocolitis, ROP- retinopathy of prematurity, IVH- intraventricular hemorrhage, PVL- periventricular leukomalacia.

Cytokine/Chemokine Abbreviations: BAFF- B-cell activating factor, BLC -B lymphocyte chemoattractant, CXCL13- CXC motif chemokine ligand 13, CD- cluster differentiating antigen, CD40L- cluster differentiating antigen 40 ligand, ENA-78- Epithelial-derived neutrophil-activating peptide 78, CCL- chemokine (C-C motif) ligand, FGF- fibroblast growth factor, Fractalkine-CX3CL1: chemokine (C-X3-C motif) ligand 1, G-CSF: Granulocyte colony stimulating factor, GM-CSF- Granulocyte-Macrophage colony stimulating factor, Gro-alpha- Growth related oncogene-alpha, HGF- Hepatocyte growth factor, ITAC- Interferon-inducible T-cell alpha chemoattractant, IFN- interferon, IL- interleukin, IP- interferon gamma-induced protein, CTLA- cytotoxic T lymphocyte-associated antigen, LIF- Leukemia inhibitory factor, M-CSF- Macrophage colony-stimulating factor, MCP- Monocyte chemoattractant protein, MDC- Macrophage-derived chemokine, MIF- macrophage migration inhibitory factor, MIP-Macrophage inflammatory protein, MMP- matrix metalloproteinase, NGF- nerve growth factor, SCF- stem cell factor, SDF- stromal cell-derived factor, TNF- tumor necrosis factor, TNF-RII - Tumor necrosis factor receptor 2, TRAIL- TNF-related apoptosis-inducing ligand, TSLP- Thymic Stromal Lymphopoietin, TWEAK- Tumor necrosis factor-like weak inducer of apoptosis, VEGF- Vascular endothelial growth factor.

XI. Supplementary table 4. List of organisms identified from blood in the culture-positive sepsis group.

	**Organism isolated from the blood in CP group**
1	CONS
2	CONS
3	CONS
4	GBS
5	CONS
6	CONS
7	Enterobacter cloacae complex;<br>Enterobacter hormaechi;<br>Klebsiella pneumoniae
8	Escherichia coli
9	Klebsiella oxytoca
10	Serratia Marcescens
11	CONS
12	Serratia Marcescens
13	CONS
14	CONS
15	GBS
16	Enterococcus faecalis
17	CONS
18	Enterococcus faecalis
19	CONS
20	CONS
21	Aspergillus niger

Supplementary table 4 foot note: CONS-Coagulase negative staphylococcus, GBS- group B streptococcus
